# Application and evolution of design in oral health: A systematic mapping study with an interactive evidence map

**DOI:** 10.1111/cdoe.12892

**Published:** 2023-08-01

**Authors:** Isobel Leason, Nicholas Longridge, Farnaz Nickpour

**Affiliations:** ^1^ Division of Industrial Design, School of Engineering University of Liverpool Liverpool UK; ^2^ School of Dentistry University of Liverpool Liverpool UK

**Keywords:** design, evidence mapping, interdisciplinary, systematic map

## Abstract

**Objectives:**

There is increasing recognition of the value and capabilities of design in healthcare. Beyond the development of medical devices, design is increasingly being applied to intangible, complex and systemic healthcare problems. However, there is limited evidence on the use of design specifically in the field of oral health. This systematic mapping study aims to collate and catalogue evidence of design in oral health.

**Methods:**

A systematic search of academic databases and grey literature was performed. Duplicate results were removed, and publications relating to the same project were grouped. Reviewers from design and oral health independently screened a sample of the dataset. Projects of both relevance to oral health, and with input from a designer or clear implementation of a design methodology or approach were included. Projects were coded and plotted on a novel interactive evidence map.

**Results:**

119 design and oral health projects were included between 1973 and 2022. Interventional (*n* = 94, 79%), empirical (*n* = 46, 39%), methodological (*n* = 35, 29%) and theoretical (*n* = 7, 6%) design contributions were identified across the projects. The projects were categorized by four orders of design: first—graphics (*n* = 6, 5%), second—products (*n* = 41, 34%), third—interactions (*n* = 70, 59%), and fourth—systems (*n* = 2, 2%). Design was found in a diverse range of contexts in oral health; most commonly being relevant to general patients (*n* = 61, 51%), and for use in general dental practice (*n* = 56, 47%). Further design outcome categories (digital material; printed material; object; room or space; apparel; process; smart device; tangible interface; graphical interface; virtual reality; service; policy; system) and oral health themes (oral health literacy; oral care training; dental clinic design; dental instruments and equipment; personal oral care; dental appliance; clinician health and productivity; clinical information systems; informed consent; oral health promotion and prevention; oral care training; patient interactions and experience) were identified.

**Conclusions:**

The novel interactive evidence map of design in oral health created enables ongoing and open‐ended multivariant documentation and analysis of the evidence, as well as identification of strategic opportunities. Future research and policy implications include; recognition and engagement with the full capabilities of design; integration of design experts; fostering inclusive engagement and collaboration; disentangling patient and public involvement; advancing human‐centred systems approaches; adopting design‐led approaches for policy‐making.

## 1 INTRODUCTION

There is increasing recognition of the strategic value and capabilities of design in healthcare. Innovation and design organizations such as NESTA^1^ and The Design Council^2^ have highlighted design's creative participatory approaches and divergent modes of thinking as beneficial for addressing healthcare challenges. There is increasing academic discourse around design for health,^3^, ^4^, ^5^, ^6^, ^7^, ^8^, ^9^, ^10^, ^11^ as well as recognition from health‐related organizations such as Wellcome,^12^ Unicef,^13^ and The Bill and Melinda Gates Foundation,^14^ and consulting and technology companies including Mckinsey & Company^15^ and IBM.^16^ Design's capacity in health spans beyond the implementation of advanced technology and development of medical devices. It is increasingly being recognized by, and integrated into healthcare organizations as a central agent of innovation and strategy,^17^, ^18^ tackling complex problems,^19^ and shaping healthcare systems and processes with a view to transition towards more desirable sustainable futures.^20^

In the context of oral health, it has been argued that design could play a valuable role in: delivering person‐centred and preventive models of care^21^; responding to socio‐demographic shifts^21^; effective adoption of technology^21^; and the translation of evidence into clinical dental practice.^22^, ^23^ However, despite the wider design and health discourse being well‐established and growing, there is limited evidence on both the use and understanding of design in oral health.

This systematic mapping review aims to—for the first time—collate and catalogue evidence of design activity in oral health, building a robust foundation to understand the current landscape and identify future opportunities for the field. To allow rigour and thoroughness of evidence mapping, each design project has been assessed according to both its design characteristics and oral health context and accordingly classified across eight categories. These include year; design contribution; design order; design outcome; oral health theme; population; setting; and collaborators.

### 1.1 Definition of design

Definitions, processes and outputs of ‘design’ vary substantially across different sectors and contexts.^24^ As such, it is important to clarify the meaning of design adopted in this review. This is influenced by the positionality of the design researchers involved, whose understanding of design is human‐centred,^25^ eurocentric^26^ and informed by their product/service design background.

This review focuses on design as a professional practice in which designers gain considerable knowledge, skills and training.^27^ We define design as a process of both problem framing and solving,^28^ which employs a combination of designerly principles, mindsets, practices and techniques,^29^ which generates outcomes across four orders of design (first—visuals, second—products, third—services and interactions, fourth—systems),^21^, ^30^ and which provides four types of contributions, that is, theoretical; methodological; empirical; as well as interventional (through design outcomes).^31^, ^32^

### 1.2 Definition of oral health

We refer to oral health as defined by the FDI^33^:

Oral health is multi‐faceted and includes the ability to speak, smile, smell, taste, touch, chew, swallow and convey a range of emotions through facial expressions with confidence and without pain, discomfort and disease of the craniofacial complex (head, face, and oral cavity). Oral health means the health of the mouth. No matter what your age, oral health is vital to general health and well‐being.

## 2 METHODOLOGY AND METHODS

This review employs a systematic mapping methodology.^34^ This type of literature review is appropriate where there is a diversity of literature and a large number of included publications is anticipated. A systematic search of a broad area of enquiry is carried out with the purpose of collating, describing and cataloguing evidence in order to identify knowledge clusters, gaps and/or define opportunities for future research, rather than to answer a focused research question. Formal quality appraisal is not required, and synthesis is presented in a clear visual format.^35^

### 2.1 Search strategy

Databases were selected based on health or design subject areas, or because of their multidisciplinary coverage. These were:

- Compendex via Engineering Village
- JSTOR
- Pubmed
- SAGE Journals
- Scopus
- Taylor and Francis Online
- Web of Science.

Titles, abstracts and keywords were searched to identify evidence of design activity in oral health up to and including December 2022.

The term ‘design’ is common in published literature. As such, a strategic search string was required to discriminate professional design practice and research. Search terminology from Chamberlian et al.'s previous review of design in health^7^ was adapted and added to by the research team and refined through pilot scoping searches. The final search terms are shown in Table 1. The search query used for each database is provided in Appendix A.

**TABLE 1:** Search terms. The search string uses Boolean operators, where the columns are combined with ‘AND’ and terms within a column with ‘OR’. Terms used for the Google search are highlighted in bold.

Terms relating to oral health		Terms relating to design	
Oral health*	Design*	‐cent*red design	**Inclusive design**
Oral care		Co‐creation	Industrial design
**Dental**		**Co‐design**	Interactive design
Dentistry		Collaborative design	Open design
		Communication design	Participatory design
		Co‐production	Policy design
		Co‐research	Practice‐based design
		Creative practice	Practice‐led design
		Critical artefact*	**Product design**
		Cultural probe*	**Service design**
		Design for all	Speculative design
		Design for policy	Storyboard
		Design probe*	**Systemic design**
		**Design thinking**	Systems approach
		Diasab*	Systems thinking
		Ergonomic*	UI design
		Evidence‐based design	Universal design
		**Experience‐based design (EBD)**	User involvement
		**Graphic design**	User journey
		Human factors	UX design

Grey literature searching and snowballing of references from the publications already included was carried out in order to support and understand gaps in the database review. A Google search^7^ was carried out using a reduced version of the database search string (bold text in Table 1).

Search results from all sources were combined, and duplicates were removed.

### 2.2 Data screening

**FIGURE 1:**
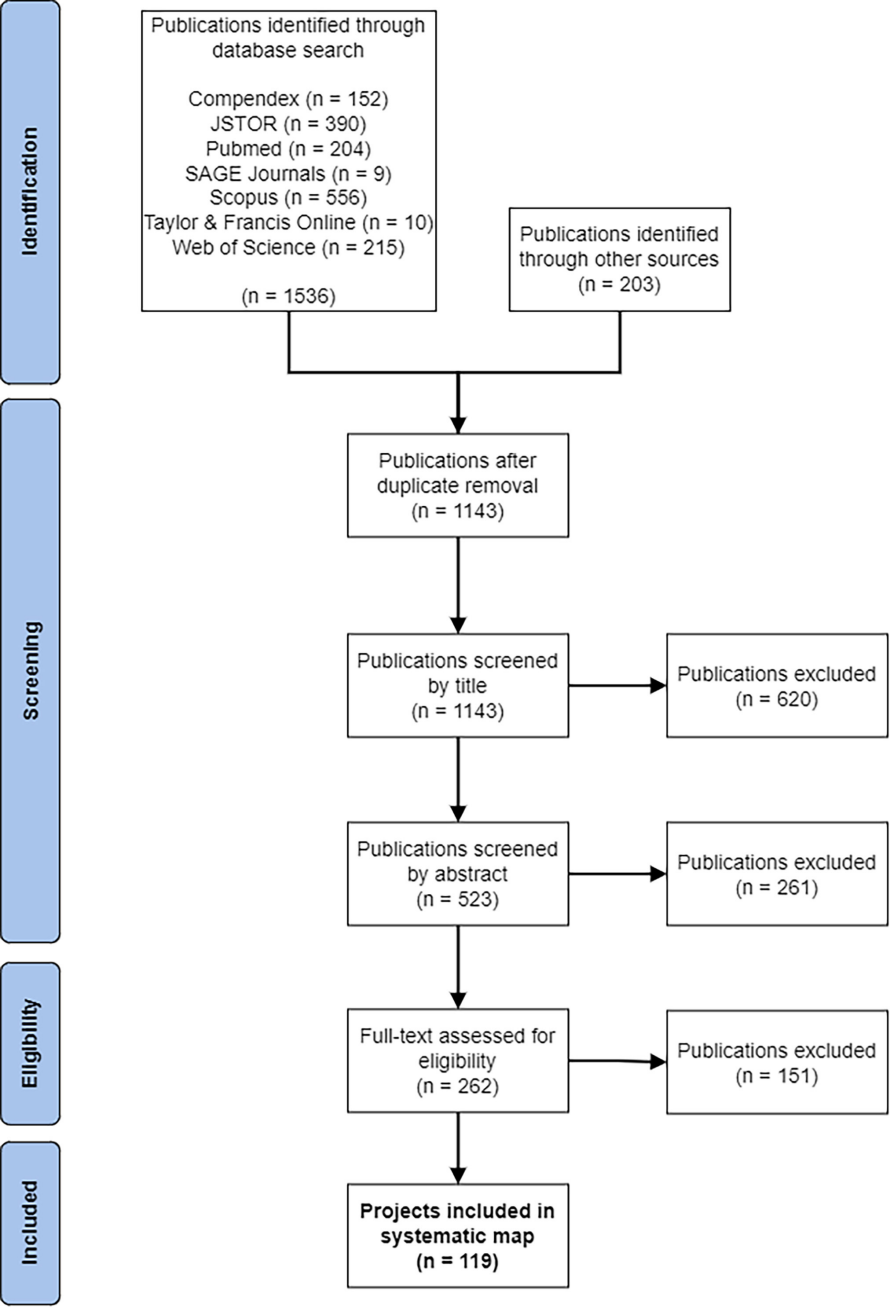
PRISMA flow diagram.

A three‐stage screening approach (title, abstract and authors, full text) was adopted. This is shown in Figure 1.

‘Projects’ are the primary unit of analysis in this review; where the search identified multiple publications related to the same project, they were grouped during the screening process.^36^

A single reviewer conducted the screening process. A multidisciplinary review team (two dental professionals and two design professionals) independently applied the screening criteria to a sample of search results (*n* = 400) to ensure consistency and clarity. There was 96% overall agreement, and the Krippendorff alpha^37^ score was 0.94 indicating substantial agreement. Discussion of any discrepancies followed, and agreements were sought to strengthen the consistency in the remaining search results.

Projects were included if both:

1. There was a contribution from the field of design.
2. The project was directly relevant to the field of oral health.

Projects were excluded if the full text was unavailable, or there was no English language version available. Literature reviews and opinion/commentary pieces were excluded. Detailed screening criteria, accompanied by example decisions, are provided in Appendix B.

### 2.3 Data extraction and analysis

Coding categories (Appendix C) were initially defined a priori (deductive approach), and were adjusted where appropriate during the coding process (inductive approach). New categories were identified during data synthesis and were incorporated into the coding scheme. Generic fields were taken from James et al's systematic mapping methodology (title, year)^34^ and topic‐specific categories were adapted from Chamberlain et al's previous design in health review (collaborators, population, setting, design output).^7^ Additional categories were added to classify design contribution, design order and oral health theme.

### 2.4 Data synthesis

The coded projects were explored through an iterative visual mapping process (Appendix E).^38^ This involved reading and re‐reading the data and exploring patterns and relationships between the projects through visual maps. This served as a creative method of synthesis, facilitating exploration of the overall landscape of design in oral health and leading to the development of an interactive evidence map (Figure 3).

## 3 RESULTS

**FIGURE 2:**
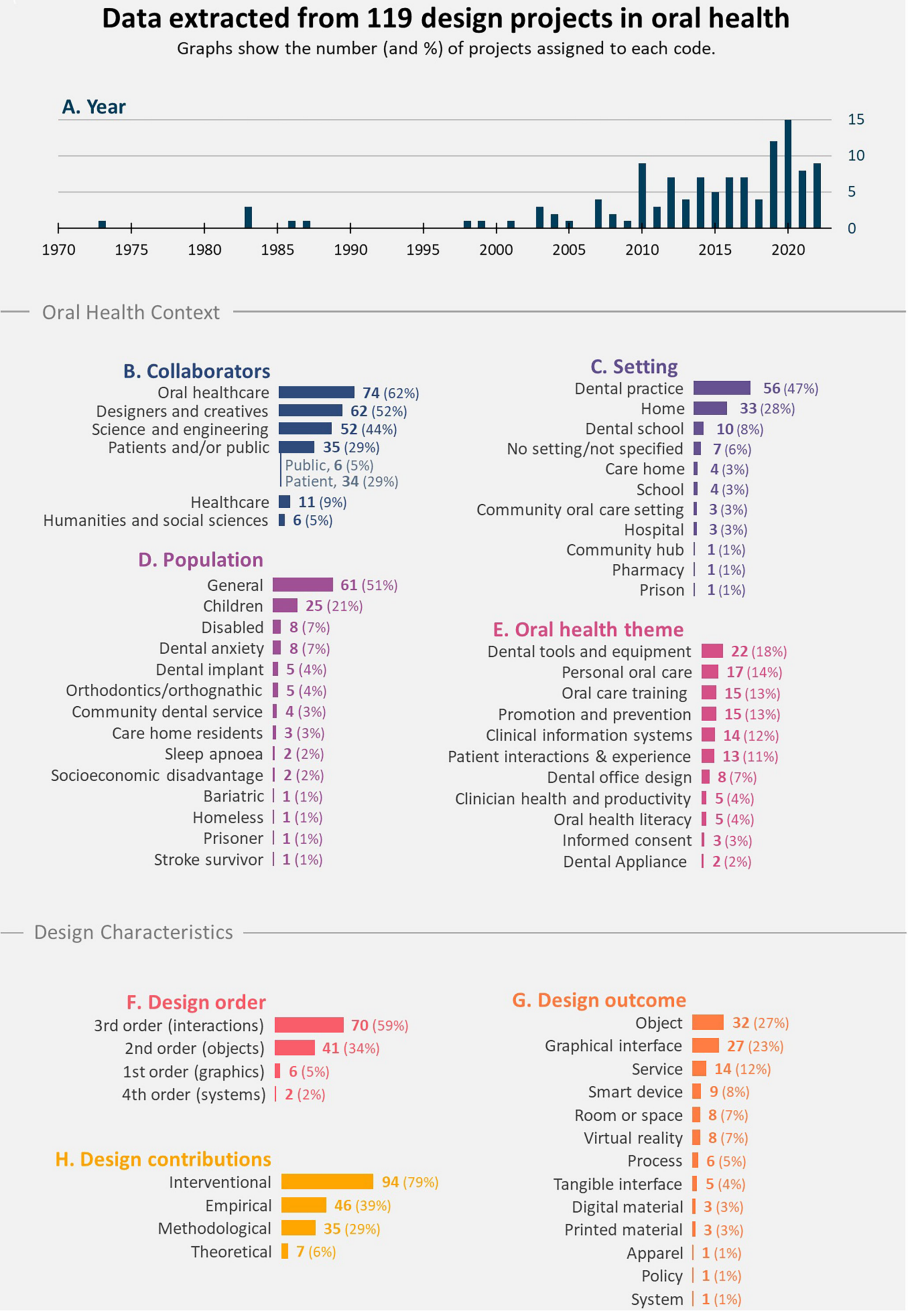
Data extracted from design projects in oral health across eight coding categories.

119 projects relating to design in oral health were identified from 1973 to 2022. The full list of coded projects can be found in Appendix D. Figure 2 illustrates the results from all coding categories to build an overall picture of the evidence.

**FIGURE 3:**
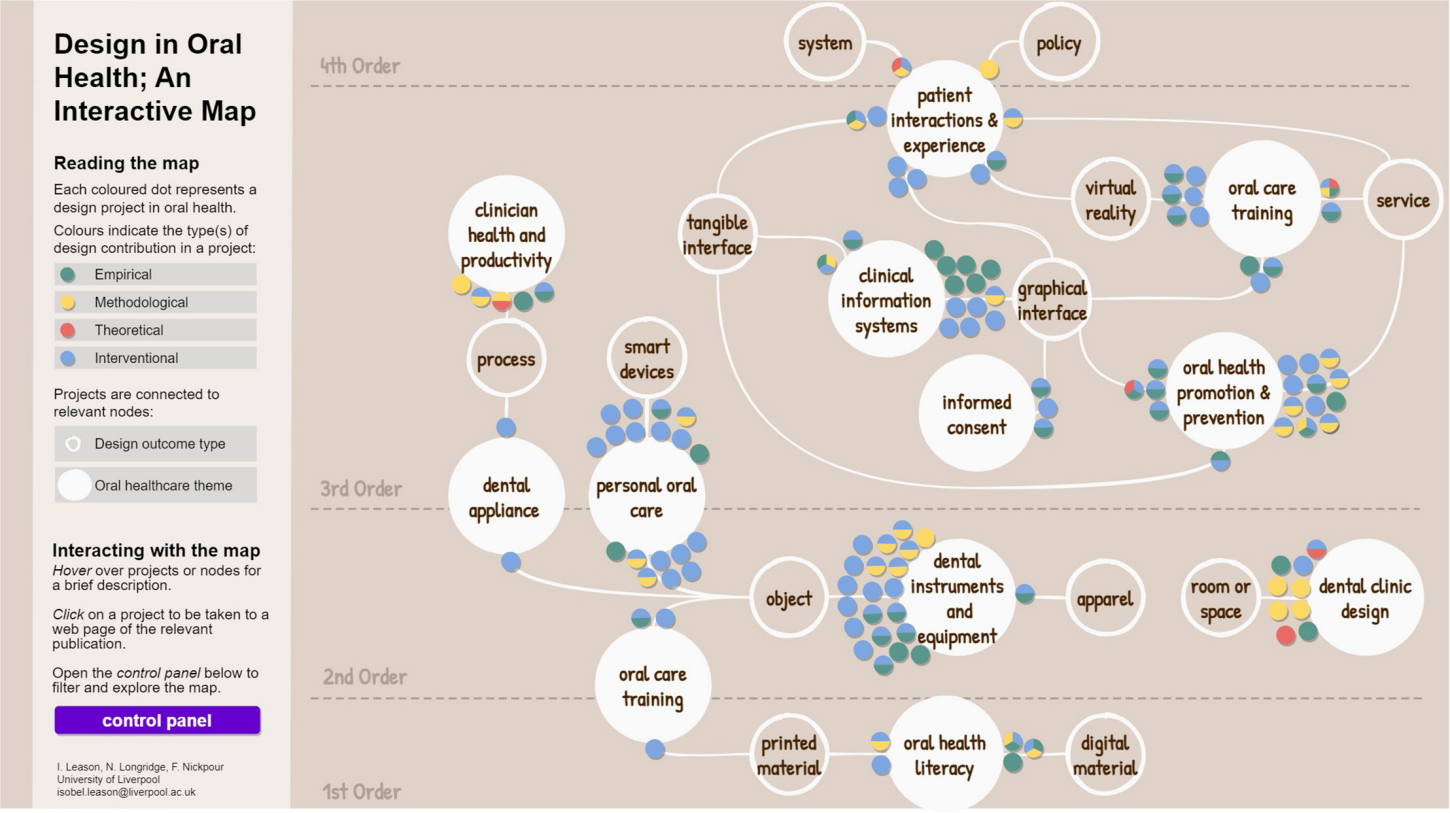
Interactive evidence map of design in oral health (available at: https://inclusionaries.com/portfolio/map‐design‐oral‐health/).

**FIGURE 4:**
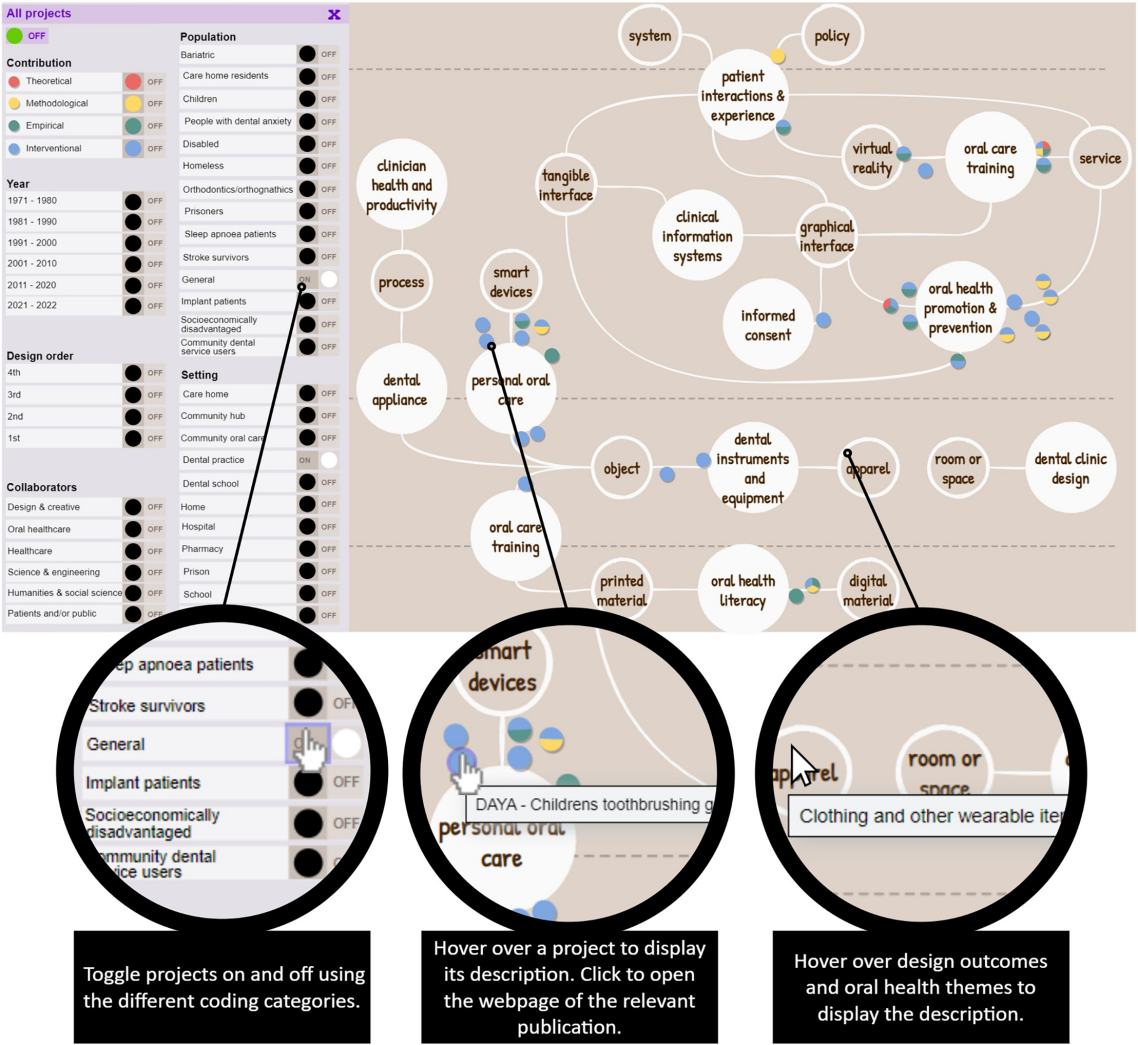
Interactive features of the evidence map.

Figure 3 shows the interactive evidence map produced and Figure 4 highlights its interactive features. Each project appears on the map as a coloured dot, plotted according to the eight design and oral health coding categories. The map can be accessed online,^39^ where users can filter projects on and off according to the different categories. Other interactive features include hovering over projects to display their description and clicking on them to be taken to the webpage of the relevant publication.

## 4 DISCUSSION

The interactive map enables open‐ended multivariant analysis of the evidence of design in oral health (based on 119 coded projects between 1973 and 2022) across multiple temporal, contextual and thematic levels spanning eight categories. To fully engage with the findings and benefit from the interactive features of the map, we encourage readers to access the online version.^39^

### 4.1 Design's characteristics in oral health; design orders, outcomes and contributions

Figure 2A shows growing design activity in oral health, with a notable increase from the early 2000's. This growth is mainly associated with technology‐driven third‐order design activity. While third‐order design encompasses the design of interactions both between people (services, processes) and between people and technology (interfaces), the latter is most dominant in oral health. The first instances of third‐order design involve the interface design of clinical information systems in 2003.^40^, ^41^ Since then, a wave of third‐order projects has emerged, with the majority (69%) having a technology‐related design outcome (tangible interfaces, graphical interfaces, smart devices and VR). As healthcare transitions towards a smart and connected system, technology will play a crucial role.^42^, ^43^ In oral health, with emerging concepts such as ‘Dentistry 4.0’,^44^ design could play a key role in ensuring that new technologies are not only functional but also human‐centred.^21^ Equally valuable, yet currently less common, is the application of 3rd‐order design to services, where it can aid shifts towards person‐centred care (e.g. Walji et al.'s^45^ human‐centred dental discharge summary).

The second most prevalent is second‐order design. This is not surprising given that this is the traditional domain of design, and the delivery of oral healthcare requires many tangible objects and physical spaces. Objects make up 73% of second‐order design, and most objects (67%) relate to dental instruments and equipment (the most common oral health theme). These include dental chair redesigns,^46^, ^47^, ^48^, ^49^, ^50^, ^51^ a redesigned dental drill,^52^ and a surgical tool vending machine.^53^

One notable finding is the scarcity of first‐order design. Despite our searches only identifying six first‐order design projects, graphic design is prevalent across oral healthcare in the form of logos, posters and packaging. Perhaps in many cases, the value or presence of first‐order design is overlooked, and thus, it may not be documented or uncovered by our search process. Moreover, it is worth noting that although there are not many isolated examples of first‐order design, higher‐order design often encompasses the orders below. For example, Nanjappa et al.^54^ describe the co‐design of Chatterbox, a toolkit to aid communication between Dental Health Support Workers and socioeconomically disadvantaged families. Although this project represents 3rd‐order service design, it also incorporates second and first order design elements, as evidenced by the design of a box and activity cards.

The lack of activity in the 4th order of design is less surprising given that the application of design to systems and policy is an emerging practice.^55^ We identified two projects that qualified as 4th‐order design; Chen and Li^56^ proposed a user‐centred oral health system for China, while Lievesley and Wassall^57^ discuss a visualization of UK health service provision used to bring a person‐centred perspective to policy‐making for community dental services. These projects occurred relatively recently—2020 and 2015. There is an increasing role for 4th‐order design in oral health, as discourse around wicked and persistent problems in healthcare grows,^58^, ^59^, ^60^ and it is argued that these can only be addressed through the integral transformation of whole health systems.^20^, ^61^, ^62^ Areas of design research including ‘transition design’^20^ and ‘design for policy’^63^ are responding to this.

Examining the orders of design offers valuable insights into the nature of design solutions in oral health. However, it is helpful to recognize that design's contributions extend beyond solutions or interventions and also encompass valuable empirical, methodological and theoretical dimensions. The empirical contributions identified demonstrate that design can aid in problem framing, for example in a study analysing human factors issues and patient perspectives on the dental photography work cycle,^64^ as well as in evaluation, such as in the validation of an interactive learning environment for children with dental anxiety.^65^ Design also offers methodologies and methods which are being applied in oral health, such as the use of co‐design to develop an oral health animation^66^ and theories and frameworks such as the application of persuasive design principles to the design of an intervention for child dental anxiety.^65^ While the systematic mapping identified examples across all contribution types, Figure 2H shows that interventional design contributions are the most common, occurring in 79% of the projects, while empirical (39%), methodological (29%), and theoretical (6%), are much less common. The skew towards interventional contributions suggests a conventional focus on design as an agent of problem‐solving, and the paucity of theoretical contributions in particular indicates insufficient theory development which is an issue in design highlighted by several others.^67^, ^68^ As design activity and applications grow in oral health, particularly into the 4th‐order of design, there is a need for an increased and enhanced knowledge basis (theory, methodology and methods) to support this.^69^ For example, design theories and methodologies for systems transitions^70^ and policy‐making^71^ could be adapted and applied in oral health.

### 4.2 Oral health contexts of design; oral health themes, settings, populations and collaborators

The dental practice is the predominant setting (47%) for design in oral health (Figure 2C). Within the dental practice, most projects relate to the themes: dental instruments and equipment, dental clinic design, and clinical information systems. The second most common setting is ‘home’ (28%), where the most common themes are personal oral care and oral health promotion and prevention. Together, these two settings account for 75% of the projects. Despite this, there is growing attention paid to design for oral health in a range of other settings such as pharmacies,^72^ community hubs^66^ and schools.^73^, ^74^, ^75^, ^76^ The oral health promotion and prevention theme encompasses the broadest range of settings compared to all other themes. The WHO^77^ and Health Education England^78^ have increased focus on oral health beyond dental practice, driven by the need to equip the population in relation to self‐care and appropriate use of dental care across the life course. Shifting towards community‐based approaches has the potential to prevent oral diseases through personalized and accessible action at individual, community and societal levels. The Whole Mouth Health project demonstrates this, through co‐designing with a range of people in different contexts and settings to develop tailored oral health resources.^79^

The population code captures the specific populations or patient groups affected by design in oral health. This might not necessarily be the end‐user of an intervention or the audience of a contribution, but the population to which it is relevant. For example, the intended user of Reynolds and Liu's^52^ dental drill is dentists, however, the relevant population is children with dental anxiety. While 51% of the projects included relate to the general population, 13 populations outside of the mainstream were identified (Figure 2D). The most common populations (after general) are children, disabled people, and people with dental anxiety. Projects for these groups often focus on tailored oral health literacy and promotion (e.g. educational app for children with dental anxiety),^80^ and the design of accessible dental clinics, equipment and personal oral care (e.g. dental chair for wheelchair users).^50^ Designing with diverse and often excluded populations is a key principle of inclusive design,^81^ which aims to bring them into the mainstream and create innovative solutions that benefit all. The potential value and significance of inclusive design approaches to current challenges and transitions in oral health has previously been argued.^21^

Due to the variety of professional titles used across disciplines, six broad codes were chosen to synthesize the collaborator types (Figure 2D). Design in oral health is becoming increasingly collaborative, often including members from a combination of oral healthcare, design, engineering and scientific disciplines. However, it commonly occurs without a designer or creative professional (48%), and where a designer is involved, their level of contribution varies. The application of ‘design without designers’^82^ can lead to misrepresentations of the discipline and tokenistic design practices which risk the loss of design's value, reach and impact. For example, in Tobias and Spaniers’^83^ publication ‘Developing a Mobile App (iGAM) to Promote Gingival Health by Professional Monitoring of Dental Selfies: User‐Centered Design Approach’, the term ‘user‐centered design’ does not appear at any point in the full text, despite being stated in the title.

Collaborators from the humanities and social sciences are least common, being identified in 5% of the projects. As design challenges in oral health become increasingly complex, involving a broad range of disciplines and stakeholders can foster co‐creativity and develop new transdisciplinary approaches. In particular, the humanities and social sciences could offer valuable perspectives on the complex social, psychological, cultural and historical contexts for design in oral health.^84^

Patients and/or the public were only involved in 29% of the projects (29% patients, 5% public), with the interactive evidence map showing increasing numbers of projects involving patients and/or the public after 2010. Public and patient collaboration is highly relevant to the calls for patient‐centred care in dentistry,^85^, ^86^ and to public and patient involvement (PPI) which is increasingly lauded and is often a requisite for securing health research funding.^87^ Design offers a variety of creative, critical and empathic participatory methods and approaches which are being applied to PPI in oral health, covering topics such as; the design of dental discharge summaries^45^ and the development of a prevention service model for low‐income communities.^88^

Adopting a design approach, we intentionally report the figures of patient and public collaborators separately, as there exists a critical distinction from a human‐centred design perspective. Human‐centred design places the needs and desires of people at the centre of the design process^25^ through engaging two distinct groups, that is, end‐users (who will interact directly with the design and have experiential knowledge), and stakeholders (who may be affected by or have an interest in the design) for different purposes and through different methods. Similar to end‐users and stakeholders, patient and public collaborators have distinct knowledge, experiences, needs and desires, and their involvement in research serves different purposes and requires different approaches. Healthcare researchers have also criticized the catch‐all term PPI,^89^ pointing out the distinct ‘meanings’ and ‘justifications’ of the two approaches,^90^ arguing that lack of clarity leads to inappropriate or tokenistic involvement.^89^ While different authors unpack the distinctions in different ways, key differences in impartiality,^89^, ^90^, ^91^ experiential knowledge,^89^, ^91^ interests,^91^ perspectives^91^ and expectations^91^ have been discussed, and there is agreement on the need to disentangle patient and public involvement.

The levels of participation of public and patient collaborators varied greatly across the projects; from evaluative user surveys, to rich involvement and collaboration throughout the design process. The need to move participation in design in oral health beyond ‘doing to/for’ and towards ‘doing with’ has previously been discussed.^82^ In order to ensure considered and meaningful involvement of patients and the public in design in oral health going forwards, we suggest making a clear distinction between public and patients and carefully considering the purpose and objectives of involvement. This will inform decisions about who to involve and how best to involve them.

### 4.3 Limitations

While we have taken a systematic and rigorous approach to identifying evidence of design in oral health, it is likely that some evidence is not reported or has been missed. Documentation and reporting standards of design have been criticized in the literature.^92^, ^93^ Design processes are generative and responsive in nature, often making them incompatible with standardized scientific methods and documentation practices. Ill‐defined and ubiquitous design terminology and limited documentation and dissemination of design practice, makes the identification and synthesis of design literature difficult. Furthermore, while methods were employed to identify grey literature, conducting an exhaustive grey literature search has inherent limitations,^94^ and data were primarily retrieved from academic literature. Academic publishing is prone to publication bias with a preference for novelty, meaning that the methodology may capture the state of the art in design, but lack representation of the status quo. Furthermore, searches were carried out in the English language, meaning that the findings are likely eurocentric and not representative of the nature of design across all geographies and cultures.

### 4.4 Implications for future design in oral health

The interactive evidence map enables ongoing and open‐ended multivariant documentation and analysis of the evidence of design in oral health as a living map. We propose that it could be used for three distinct purposes;

1. A documentation tool—to capture and map chronological evolution of the field.
2. An analytical tool—to facilitate in‐depth exploration and multivariate analysis of evidence of design in health, enabling the identification of trends across different categories/levels/contexts.
3. A strategic and generative tool—to inform new interdisciplinary research streams and collaborations through the identification of critical gaps, strategic opportunities and emerging themes at the intersection of the two fields and beyond.

Evidence of design in oral health identified through this first systematic mapping review raises multiple future research and policy implications for this interdisciplinary field, as outlined in Table 2.

**TABLE 2:** Implications for future design in oral health.

RESEARCH Implications—Design in oral health
**1. Strategic research directions and dissemination;**
Moving beyond individual disjointed projects, investigate critical gaps and key opportunities arising from the current landscape, and advance strategic collaborative research agenda and directions at the frontier of design in oral health. Develop effective dissemination strategies to ensure that design approaches, contributions and outcomes are shared and implemented widely and at scale.
**2. Recognition of and engagement with the full capabilities of design;**
[2.1] In both processes of: problem framing and problem‐solving.
[2.2] Across all four design contribution types: theories, methodologies, empirical studies, and interventions or solutions.
[2.3] In generating a range of outcomes across all four orders of design: 1st graphics, 2nd objects, 3rd interactions, 4th systems.
**3. Integration of design experts;**
Ensure core involvement and leadership of design experts in any design‐related activity from the outset and throughout, and avoid ‘design without designers’.
**4. Fostering inclusive engagement and collaboration;**
Engage and convene a broad range of disciplines and stakeholders and develop transdisciplinary approaches for design in oral health. Invite diverse less hierarchical perspectives and actively identify and collaborate with typically marginalized and excluded voices.
**5. Advancing human‐centred systems approaches;**
Leverage ‘individual‐level’ experiences with the wider ‘system‐level’ interconnected factors to address complex problems in oral health. This involves disentangling patient and public collaboration, and considering community‐based and tailored approaches to address the unique needs of different populations.
**POLICY Implications—Design in oral health**
**6. Design for oral health policy‐making;**
Explore and adopt ‘design for policy’; design‐led approaches for systematically developing effective human‐centred policies based on leveraging creative participatory approaches, evidence‐based criteria, and novel concepts.
**7. Strategic research funding;**
Target key areas identified through the landscape of design in oral health with grant calls to stimulate research streams in priority areas.
**8. Disentangle patient and public involvement;**
Develop a granular, human‐centred design approach to public and patient involvement, which makes a distinction between patients and the public. Emphasize clarifying the purpose and objectives of involvement to inform decisions about who to involve and how best to involve them.

## FUNDING INFORMATION

Funded by University of Liverpool Doctoral Network for Technologies for Healthy Ageing.

## CONFLICT OF INTEREST STATEMENT

The authors have no conflicts of interest to declare.

## Supporting information


**Appendix S1.** Supporting Information

## Data Availability

The data that supports the findings of this study are available in the supplementary material of this article
