# Supplementary material for: Application and evolution of design in oral health: A systematic mapping study with an interactive evidence map
Source: Community Dent Oral Epidemiol. 2023 Aug 1;52(1):1–12. doi: 10.1111/cdoe.12892 (PMC10952138; doi:10.1111/cdoe.12892)

**Appendices**

**Appendix A: Search Queries**

**Appendix B: Screening Criteria and Example Decisions**

**Appendix C: Coding Categories**

**Appendix D: Coded Projects**

**Appendix E: Visual Mapping Process**

# 1. Appendix A: Search Queries

Appendix A contains a record of the search query used in each database. Where fields could be added to the search query title/abstract/keyword searching was used if available, if not title/abstract was used, and if that wasn’t available abstract searching was used.

## 1.1. Compendex via Engineering Village

(((("oral health*" OR "oral care" OR "dental" OR "dentistry") WN KY) AND (("design*") WN KY)) AND (("-centred design" OR "-centered design" OR "Co-creation" OR "Co-design" OR "Collaborative design" OR "Communication design" OR "Co-production" OR "Co-research" OR "Creative practice" OR "Critical artefact*" OR "Cultural probe*" OR "Design for all" OR "Design for policy" OR "Design probe*" OR "Design thinking" OR Diasab* OR Ergonomic* OR "Evidence-based design" OR "Experience-based design" OR "Graphic design" OR "Human factors" OR "Inclusive design" OR "Industrial design" OR "Interactive design" OR "Open design" OR "Participatory design" OR "Policy design" OR "Practice-based design" OR "Practice-led design" OR "Product design" OR "Service design" OR "Speculative design" OR Storyboard OR "Systemic design" OR "Systems approach" OR "Systems thinking" OR "UI design" OR "Universal design" OR "User involvement" OR "User journey" OR "UX design") WN KY))

## 1.2. JSTOR

Due to JSTOR’s 200-character limit the search was split up into nine searches. Only abstracts were searched, as JSTOR doesn't have a title, abstract, keyword searching function.

*search 1:*

abs:("oral health*" OR "oral care" OR "dental" OR "dentistry") AND abs:("design*") AND abs:("-centred design" OR "-centered design" OR "Co-creation" OR "Co-design" OR "Collaborative design")

*search 2:*

abs:("oral health*" OR "oral care" OR "dental" OR "dentistry") AND abs:("design*") AND abs:("Communication design" OR "Co-production" OR "Co-research" OR "Creative practice" OR "Critical artefact*")

*search 3:*

abs:("oral health*" OR "oral care" OR "dental" OR "dentistry") AND abs:("design*") AND abs:("Cultural probe*" OR "Design for all" OR "Design for policy" OR "Design probe*" OR "Design thinking")

*search 4:*

abs:("oral health*" OR "oral care" OR "dental" OR "dentistry") AND abs:("design*") AND abs:(“diasab*” OR "Ergonomic*" OR "Evidence-based design" OR "Experience-based design" OR "Graphic design")

*search 5:*

abs:("oral health*" OR "oral care" OR "dental" OR "dentistry") AND abs:("design*") AND abs:(“Human factors” OR "Inclusive design" OR "Industrial design" OR "Interactive design" OR "Open design")

*search 6:*

abs:("oral health*" OR "oral care" OR "dental" OR "dentistry") AND abs:("design*") AND abs:("Participatory design" OR "Policy design" OR "Practice-based design" OR "Practice-led design")

*search 7:*

abs:("oral health*" OR "oral care" OR "dental" OR "dentistry") AND abs:("design*") AND abs:("Product design" OR "Service design" OR "Speculative design" OR Storyboard OR "Systemic design")

*search 8:*

abs:("oral health*" OR "oral care" OR "dental" OR "dentistry") AND abs:("design*") AND abs:(“Systems approach” OR "Systems thinking" OR "UI design" OR "Universal design" OR "User involvement")

*search 9:*

abs:("oral health*" OR "oral care" OR "dental" OR "dentistry") AND abs:("design*") AND abs:( "User journey" OR "UX design")

## 1.3. Pubmed

Phrases not found in phrase index and removed by Pubmed: “critical afterfact*”, “design for policy”, “practice-led design”.

("oral health*"[Title/Abstract] OR "oral care"[Title/Abstract] OR "dental"[Title/Abstract] OR "dentistry"[Title/Abstract]) AND "design*"[Title/Abstract] AND ("centred design"[Title/Abstract] OR "centered design"[Title/Abstract] OR "Co-creation"[Title/Abstract] OR "Co-design"[Title/Abstract] OR "Collaborative design"[Title/Abstract] OR "Communication design"[Title/Abstract] OR "Co-production"[Title/Abstract] OR "Co-research"[Title/Abstract] OR "Creative practice"[Title/Abstract] OR "cultural probe*"[Title/Abstract] OR "Design for all"[Title/Abstract] OR "design probe*"[Title/Abstract] OR "Design thinking"[Title/Abstract] OR "diasab*"[Title/Abstract] OR "ergonomic*"[Title/Abstract] OR "Evidence-based design"[Title/Abstract] OR "Experience-based design"[Title/Abstract] OR "Graphic design"[Title/Abstract] OR "Human factors"[Title/Abstract] OR "Inclusive design"[Title/Abstract] OR "Industrial design"[Title/Abstract] OR "Interactive design"[Title/Abstract] OR "Open design"[Title/Abstract] OR "Participatory design"[Title/Abstract] OR "Policy design"[Title/Abstract] OR "Practice-based design"[Title/Abstract] OR "Product design"[Title/Abstract] OR "Service design"[Title/Abstract] OR "Speculative design"[Title/Abstract] OR "Storyboard"[Title/Abstract] OR "Systemic design"[Title/Abstract] OR "Systems approach"[Title/Abstract] OR "Systems thinking"[Title/Abstract] OR "UI design"[Title/Abstract] OR "Universal design"[Title/Abstract] OR "User involvement"[Title/Abstract] OR "User journey"[Title/Abstract] OR "UX design"[Title/Abstract])

## 1.4. SAGE Journals

("oral health*" OR "oral care" OR "dentistry" OR "dental") AND "design*" AND ("-centred design" OR "-centered design" OR "Co-creation" OR "Co-design" OR "Collaborative design" OR "Communication design" OR "Co-production" OR "Co-research" OR "Creative practice" OR "Critical artefact*" OR "Cultural probe*" OR "Design for all" OR "Design for policy" OR "Design probe*" OR "Design thinking" OR Diasab* OR Ergonomic* OR "Evidence-based design" OR "Experience-based design" OR "Graphic design" OR "Human factors" OR "Inclusive design" OR "Industrial design" OR "Interactive design" OR "Open design" OR "Participatory design" OR "Policy design" OR "Practice-based design" OR "Practice-led design" OR "Product design" OR "Service design" OR "Speculative design" OR Storyboard OR "Systems approach" OR "Systems thinking" OR "UI design" OR "Universal design" OR "User involvement" OR "User journey" OR "UX design")

## 1.5. Scopus

( TITLE-ABS-KEY ( "oral health*" OR "oral care" OR dentistry OR dental ) AND TITLE-ABS-KEY ( design* ) AND TITLE-ABS-KEY ( "-centred design" OR "-centered design" OR "Co-creation" OR "Co-design" OR "Collaborative design" OR "Communication design" OR "Co-production" OR "Co-research" OR "Creative practice" OR "Critical artefact*" OR "Cultural probe*" OR "Design for all" OR "Design for policy" OR "Design probe*" OR "Design thinking" OR diasab* OR ergonomic* OR "Evidence-based design" OR "Experience-based design" OR "Graphic design" OR "Human factors" OR "Inclusive design" OR "Industrial design" OR "Interactive design" OR "Open design" OR "Participatory design" OR "Policy design" OR "Practice-based design" OR "Practice-led design" OR "Product design" OR "Service design" OR "Speculative design" OR storyboard OR "Systems approach" OR "Systemic design" OR "Systems thinking" OR "UI design" OR "Universal design" OR "User involvement" OR "User journey" OR "UX design" ) )

## 1.6. Taylor and Francis Online

[[Abstract: "oral health*"] OR [Abstract: "oral care"] OR [Abstract: dental] OR [Abstract: dentistry]] AND [Abstract: design*] AND [[Abstract: "-centred design"] OR [Abstract: "-centered design"] OR [Abstract: "co-creation"] OR [Abstract: "co-design"] OR [Abstract: "collaborative design"] OR [Abstract: "communication design"] OR [Abstract: "co-production"] OR [Abstract: "co-research"] OR [Abstract: "creative practice"] OR [Abstract: "critical artefact*"] OR [Abstract: "cultural probe*"] OR [Abstract: "design for all"] OR [Abstract: "design for policy"] OR [Abstract: "design probe*"] OR [Abstract: "design thinking"] OR [Abstract: diasab*] OR [Abstract: ergonomic*] OR [Abstract: "evidence-based design"] OR [Abstract: "experience-based design"] OR [Abstract: "graphic design"] OR [Abstract: "human factors"] OR [Abstract: "inclusive design"] OR [Abstract: "industrial design"] OR [Abstract: "interactive design"] OR [Abstract: "open design"] OR [Abstract: "participatory design"] OR [Abstract: "policy design"] OR [Abstract: "practice-based design"] OR [Abstract: "practice-led design"] OR [Abstract: "product design"] OR [Abstract: "service design"] OR [Abstract: "speculative design"] OR [Abstract: storyboard] OR [Abstract: "systems approach"] OR [Abstract: "systemic design"] OR [Abstract: "systems thinking"] OR [Abstract: "ui design"] OR [Abstract: "universal design"] OR [Abstract: "user involvement"] OR [Abstract: "user journey"] OR [Abstract: "ux design"]]

## 1.7. Web of Science

((TS=("oral health*" OR "oral care" OR "dentistry" OR "dental" )) AND TS=("design*" )) AND TS=("-centred design" OR "-centered design" OR "Co-creation" OR "Co-design" OR "Collaborative design" OR "Communication design" OR "Co-production" OR "Co-research" OR "Creative practice" OR "Critical artefact*" OR "Cultural probe*" OR "Design for all" OR "Design for policy" OR "Design probe*" OR "Design thinking" OR Diasab* OR Ergonomic* OR "Evidence-based design" OR "Experience-based design" OR "Graphic design" OR "Human factors" OR "Inclusive design" OR "Industrial design" OR "Interactive design" OR "Open design" OR "Participatory design" OR "Policy design" OR "Practice-based design" OR "Practice-led design" OR "Product design" OR "Service design" OR "Speculative design" OR Storyboard OR "Systems approach" OR "Systemic design" OR "Systems thinking" OR "UI design" OR "Universal design" OR "User involvement" OR "User journey" OR "UX design")

## 1.8. Google

“oral health*” OR “dental” AND “-cent*red design” OR “co-design” OR “design thinking” OR “inclusive design” OR “experience based design” OR “graphic design” OR “product design” OR “service design” OR “systemic design”

# 2. Appendix B: Screening Criteria and Example Decisions

A three-stage screening approach was adopted. Titles were screened in the first stage, the abstract and authors were checked in the second stage, and the full text was reviewed in the third stage. The PRISMA flowchart shown in Figure 2 summarises this process.

‘Projects’ are the primary unit of analysis and interest in this review. A single project may have multiple publications about it, and each of these may contribute useful information. Therefore, where the search identified multiple publications related to the same project, they were grouped during the screening process. This is the same process as a systematic review in which multiple reports of the same study are identified and associated with each other manually by the review author.

A single reviewer conducted the screening process. Inclusion and exclusion criteria were checked for consistency and clarity by a multidisciplinary review team (two dental professionals and two design professionals). Each member of the review team independently applied the criteria to a sample of search results (n=400). The krippendorff alpha statistic was used to assess intercoder reliability. There was 96% overall agreement, and the Krippendoff alpha score was 0.94 indicating substantial agreement. Discussion of the discrepancies in inclusion decisions followed, and agreements were sought to strengthen the consistency in the remaining search results.

Projects were included if both:

1. There was a contribution from the field of design.

*Projects were included if a design professional was involved, or there was clear implementation of design principles, practices, mindsets, or techniques.*

1. The project was directly relevant to the field of oral healthcare.

*A loose criterion for oral healthcare was adopted. Projects relating to oral health (as defined by the FDI^1^) or the direct provision of oral care were included; this includes dental education, equipment, public health, care pathways and policy.*

Projects were excluded if the full text was unavailable, or there was no English language version available. Literature reviews and opinion/commentary pieces were excluded.

## 2.1. Screening criteria

*Table 1. Inclusion and exclusion criteria*

| **Inclusion**  ***Both I.1 and I.2 must be true to be included.*** | | **Exclusion**  ***A project will be excluded if any of E.1-4 are true.*** | |
| --- | --- | --- | --- |
| I.1 | There is a contribution from the field of design. | E.1 | Full text not available. |
| I.2 | The project is directly relevant to the field of oral healthcare. A brief mention of potential application to the field of oral healthcare does not count. | E.2 | No english language version of the text available. |
|  |  | E.3 | The publication is a literature review or opinion/commentary piece. |
|  |  | E.4 | The text was published after 2022. |

## 2.2. Relevant definitions

**‘Design’** - for assessing design input (I.1):

Design is a professional practice. It is conducted through a design process (of framing and solving) and employs a combination of principle, practices, mindsets and techniques. Our definition of design is human-centred and involves engaging with people, creative experimentation, prototyping, gathering feedback and redesign. It can make theoretical, empirical, methodological and interventional contributions to the design across the four orders (graphics, products, services, systems). Design input is evidenced by the inclusion of a design author or by the presence of design principles, practices, mindsets or techniques e.g. co-design, user-centred design, double diamond, design thinking.

**‘Oral healthcare’** - for assessing relevance (I.2):

Our definition of oral healthcare is based upon the FDI definition of oral health:

*“Oral health is multi-faceted and includes the ability to speak, smile, smell, taste, touch, chew, swallow and convey a range of emotions through facial expressions with confidence and without pain, discomfort and disease of the craniofacial complex (head, face, and oral cavity). Oral health means the health of the mouth. No matter what your age, oral health is vital to general health and well-being.”*

FDI. FDI’s definition of oral health. Published 2016. Accessed June 16, 2021 https://www.fdiworlddental.org/fdis-definition-oral-health

In this case, oral healthcare includes anything related to oral health or the direct provision of oral care; this includes dental education, practitioner wellbeing and efficiency, equipment, public health, care pathways and policy.

## 2.3. Examples of common borderline cases with decision rationale

*Ergonomics*

The search returned lots of results related to ergonomics. Whilst ergonomics is a discipline associated with design, it should not automatically be included as a design contribution. Results mentioning ergonomics were only included if there was input from a design professional, or there was a clear design input (I.1).

An example of included and excluded literature related to ergonomics is given below for clarity.

*Table 2. Example decisions relating to ergonomics*

| **Decision** | **Publication** | **Reason** |
| --- | --- | --- |
| Included | Haddad, O. et al. (2012) ‘Trapezius muscle activity in using ordinary and ergonomically designed dentistry chairs’, international journal of occupational and environmental medicine, 3(2), pp. 76–83. | This is an ergonomic study which describes the design and evaluation of a chair. |
| Excluded | Chaiklieng, S. and Suggaravetsiri, P. (2015) ‘Ergonomics Risk and Neck Shoulder Back Pain among Dental Professionals’, Procedia manufacturing, 3, pp. 4900–4905. | This is a study of ergonomic risk and neck shoulder back pain over time. Although this could be relevant to design, nothing is designed, and there is no design input, or design principles, practices, mindsets or methods present. |

*Engineering*

The screening process highlighted a fuzzy boundary between when contributions from engineering/technology might count as design. This is a difficult crossover. It is important to recognise and include design within engineering, however, if every incidence of engineering/technology was included in the study they would greatly outnumber all other contributions and produce an unmanageable data set. Whilst design contributions can come from engineers (and some designers come from engineering backgrounds), there is a difference between the disciplines thinking and approaches. Design is a human-centred iterative process which involves creative experimentation, prototyping, gathering feedback and redesign. Engineering contributions often focus on material innovation, technology or manufacturing without consideration of the end user. They also often lack the ‘problem framing’ stage of the design process, as well as an exploratory process of iterative testing and evaluation. An agreement was made that only engineering designs where design methodologies were identified and implemented, or there was a clear process of problem framing and problem solving, or users were involved in the design process were included. Example decisions are provided below.

*Table 3. Example decisions relating to engineering*

| **Decision** | **Publication** | **Reason** |
| --- | --- | --- |
| Included | Tse, B. et al. (2010) ‘Design and Development of a Haptic Dental Training System - hapTEL’, in Haptics: Generating and Perceiving Tangible Sensations. Berlin, Heidelberg: Springer Berlin Heidelberg, pp. 101–108. | There is a framing of the problem, user requirements, and development and evaluation of multiple prototypes. |
| Excluded | Kale, P.J., Metkar, R.M. and Hiwase, S.D. (2016) ‘Development and Optimization of Dental Crown Using Rapid Prototyping Integrated with CAD’, in Advances in 3D Printing & Additive Manufacturing Technologies. Singapore: Springer Singapore, pp. 169–182. | Focus on material strength analysis. No consideration of user. |

*Informatics*

There were a number of contributions from the field of dental informatics. Informatics is linked to the fields of human-computer-interaction, interface and interaction design. It was agreed that dental informatics were included if they were concerned with the usability of, or redesigned an interface or interaction. This generally meant contributions from the realm of human computer interactions were included (i.e. considering how a person interacts with a computer interface), and those concerned with ‘back-end’ computing and data analysis were not. Example decisions are provided below.

*Table 4. Example decisions relating to informatics*

| **Decision** | **Publication** | **Reason** |
| --- | --- | --- |
| Included | Walji, M.F. et al. (2012) ‘Detection and characterization of usability problems in structured data entry interfaces in dentistry’, International journal of medical informatics (Shannon, Ireland), 82(2), pp. 128–138. Available at: https://doi.org/10.1016/j.ijmedinf.2012.05.018. | Conducted a usability study of an interface, and redesigned it using iterative design methods. |
| Excluded | Amiri N, Matthews DC, Gao Q. Designing a framework of intelligent information processing for dentistry administration data. Int J Comput Dent. 2005 Jul;8(3):221-31. English, German. PMID: 16416935. | Focus on raw data processing with no user considerations or interface design. |

# 3. Appendix C: Coding Categories

*Table 5. Coding procedure used to categorise each project.*

| **Category** | **Description** |
| --- | --- |
| Title | Short description of the project. |
| Year | Year of publication.  Where a project has multiple publications, the year of the first publication is used. |
| Collaborator(s) | The disciplines or stakeholder groups involved in producing the contribution. |
| Design contribution | Interventional, Empirical, Methodological, or Theoretical.  A project may have multiple contribution types. |
| Design order | 1st, 2nd, 3rd, or 4th.  A project can only be assigned one order as higher orders encompass those below them. |
| Setting | Where the related output will be used. |
| Population | The population or patient group that the project concerns. |
| Design outcome | The type of outcome the project is producing or is relevant to. |
| Oral health theme | The area of oral health which the project relates to. |

*Table 6. Description of codes*

| **Category** | **Code** | **Description** |
| --- | --- | --- |
| Design order | 1st order | The design of graphics and communication. |
|  | 2nd order | The design of tangible objects. |
|  | 3rd order | The design of human experiences (meaning interactions with other people and objects); including interfaces, activities and services. |
|  | 4th order | The design of complex systems. |
| Design contribution | Interventional | New or improved products, services, systems or artefacts. |
|  | Empirical | Data sets, surveys, arguments or findings which reveal formerly unknown insight and analysis of behaviours, capabilities, or interactions with interventions, etc. |
|  | Methodological | Methodologies, methods, processes or techniques. |
|  | Theoretical | Conceptual models, frameworks, policies or principles. |
| Collaborators | Oral healthcare professional | Anyone one who works in oral healthcare practice, research or policy. |
|  | Designers and creative disciplines | Any design or creative discipline including visual arts, graphic design, product design, fashion design, and web design. |
|  | Science and engineering | Disciplines such as physics, chemistry, biology, computer science, mechanical engineering, electrical engineering, and chemical engineering. |
|  | Patients and/or public | Anyone involved in the projects in a non-professional capacity.  Patient: Involved on basis of their experiential knowledge and/or role as an end user.  Public: Involved based on their impartiality, or affected the design/research indirectly. |
|  | Healthcare professionals | Anyone who works in healthcare practice, research or policy. |
|  | Humanities and social sciences | Disciplines such as sociology, anthropology, law, and psychology. |
| Setting | Dental practice | A mainstream oral healthcare facility offering primary dental care services. |
|  | Home | A persons residence. |
|  | Dental school | An educational institution where individuals can obtain professional training in the field of dentistry. |
|  | No setting/not specified | Either no specific setting is relevant to the project, or no setting is specified. |
|  | Care home | A residential facility that provides accommodation and personal care services to individuals who require assistance with daily living activities, typically due to advanced age, disability, or illness. |
|  | School | An educational institution where children and young adults receive academic instruction and participate in extracurricular activities. |
|  | Community oral care setting | A service that provides dental care to individuals in the community. This may include mobile dental units, or other community-based oral health programs. |
|  | Hospital | A healthcare facility that provides a wide range of medical and surgical services to patients with acute or chronic health conditions. |
|  | Community hub | A public space or facility that serves as a central gathering place for people in a local community. |
|  | Pharmacy | A healthcare facility that dispenses prescription medications and provides advice on the safe and effective use of medications. |
|  | Prison | A correctional facility where individuals who have been convicted of a crime are incarcerated. |
| Population | General | The overall population, without any specific demographic, medical, or social characteristic. |
|  | Children | Young individuals who have not yet reached adulthood. |
|  | Disabled people | Individuals who have a long-term health condition, impairment or illness that has a substantial effect on their daily living. |
|  | People with dental anxiety | People who experience dental anxiety. |
|  | Dental implant patients | Patients recieving dental implants. |
|  | Orthodontics and orthognathic surgery patients | Patients undergoing orthodontic treatment and/or orthognathic surgery. |
|  | Community dental service users | Patients who access community dental services. |
|  | Care home residents | People who live in a care home. |
|  | Sleep apnoea patients | Patients who are recieving treatment due to sleep apnoea. |
|  | Socioeconomic disadvantaged people | People living in less favourable social and economic circumstances than others in the same society. |
|  | Bariatric patients | Patients who are obese. |
|  | People experiencing homelessness | People who are experiencing homelessness. |
|  | Prisoners | People who are incarcerated. |
|  | Stroke survivors | People who have survived a stroke. |
| Design outcome | Object | Tangible items such as instruments, devices and furniture. |
|  | Graphical interface | A digital interface in which a user interacts with digital material through icons, buttons, menus etc. |
|  | Service | A set of activities or processes that are performed to meet a specific customer need or goal. |
|  | Smart device | Physical objects combined with digital elements which exchange data about the product/environment/users. Often accessed via an app or other digital interface. |
|  | Room or space | A defined physical area within a building created for a specific purpose. |
|  | Virtual reality | Computer-generated simulation of a three-dimensional image or environment that can be interacted with in a seemingly real or physical way. |
|  | Process | A series of actions which achieve a specific goal. |
|  | Tangible interface | Physical objects through which the user interacts with a digital interface. |
|  | Digital material | Graphics (including text, image and video) for use on digital platforms such as social media and websites. |
|  | Printed material | Graphics (including text and images) designed to be printed on a tangible surface (usually paper). |
|  | Apparel | Clothing and other wearable items. |
|  | System | An interrelated set of components (organisations, governments, stakeholders, financing etc) that interact to accomplish a defined objective. |
|  | Policy | Regulation, procedure, administrative action, incentive, or voluntary practice on a local, national or international scale. |
| Oral health theme | Dental instruments and equipment | Items used to deliver care in oral healthcare settings by oral healthcare professionals. |
|  | Personal oral care | Activities undertaken by individuals outside of oral healthcare settings to maintain good oral hygiene. |
|  | Oral health promotion and prevention | Proactive measures to maintain and improve the oral health of individuals and communities, including increasing awareness and understanding of oral health. |
|  | Oral care training | Education and training of oral health professionals. |
|  | Clinical information systems | Computer systems that provide immediate access to current patient data and facilitate direct patient care. |
|  | Patient interactions and experience | Interactions and experiences of dental patients from booking an appointment to receiving treatment. |
|  | Dental clinic design | Planning and design of the built environment, including rooms and layout, of a dental clinic. |
|  | Oral health literacy | Ensuring individuals have the capacity to obtain, process and understand health information and services in order to make appropriate health decisions. |
|  | Clinician health and productivity | Factors which affect clinicians’ ability to perform tasks including work-related ergonomics and musculoskeletal disorders. |
|  | Informed consent | Informing patients about the nature of their dental condition, the treatment options available, and the associated benefits and risks, so that they can make an informed decision about the best course of action for their specific needs. |
|  | Oral appliances | Customised oral devices to repair, straighten or replace teeth. |

# 3. Appendix D: List of Coded Projects

*Table 7. Coded projects. Listed chronologically.*

| **Project** | | **Reference** | **Year** | **Collaborator(s)** | **Design Contributions** | **Design order** | **Setting** | **Oral health patient group** | **Design output** | **Oral healthcare theme** |
| --- | --- | --- | --- | --- | --- | --- | --- | --- | --- | --- |
| **P1** | **Dental syringe, designed to improve the efficiency of the dental team in performing restorative operations.** | ^2^ | 1973 | Oral healthcare professionals | E  I | 2 | Dental practice | General patients | Object | Dental tools and equipment |
| **P2** | ***University of Mississippi Dental Care Unit*. Automatic brushing for those unable to use hands.** | ^3^ | 1983 | Oral healthcare professionals | I | 2 | Home | Disabled people | Object | Personal oral care |
| **P3** | ***MOTEC*. Mobile oral treatment and examination chair.** | ^4^ | 1983 | Oral healthcare professionals | I | 2 | Community oral care setting | Community dental service users | Object | Dental tools and equipment |
| **P4** | **Portable dental chair.** | ^5^ | 1983 | Oral healthcare professionals | I | 2 | Community oral care setting | Community dental service users | Object | Dental tools and equipment |
| **P5** | **Methods and guidelines for accessible dental office design.** | ^6^ | 1986 | Designers and creative disciplines  Oral healthcare professionals | M | 2 | Dental practice | Disabled people | Room or space | Dental office design |
| **P6** | **Dentist's perceptions of dental office design.** | ^7^ | 1987 | Designers and creative disciplines  Oral healthcare professionals | E | 2 | Dental practice | General patients | Room or space | Dental office design |
| **P7** | **Ergonomic recommendations and methods for dental chair design.** | ^8^ | 1998 | Designers and creative disciplines | M | 2 | Dental practice | General patients | Object | Dental tools and equipment |
| **P8** | **Interior design for dentistry.** | ^9^ | 1999 | Designers and creative disciplines | M | 2 | Dental practice | General patients | Room or space | Dental office design |
| **P9** | ***Waterpik.* Water flosser.** | ^10^ | 2001 | Designers and creative disciplines | I | 2 | Home | General patients | Object | Personal oral care |
| **P10** | ***ORQUEST.* IT support for chairside work in dentistry.** | ^11^ | 2003 | Oral healthcare professionals  Science and engineering disciplines | I | 3 | Dental practice | General patients | Graphical interface | Clinical information systems |
| **P11** | **Speech and gesture interaction with clinical interfaces.** | ^12–15^ | 2003 | Science and engineering disciplines  Oral healthcare professionals | M  E  I | 3 | Dental practice | General patients | Tangible interface | Clinical information systems |
| **P12** | ***Adele.* Pedagogical agent.** | ^16^ | 2003 | Science and engineering disciplines  Oral healthcare professionals | I  E | 3 | Dental school | General patients | Graphical interface | Oral care training |
| **P13** | **Commercial toothbrush research and development.** | ^17^ | 2004 | Designers and creative disciplines  Science and engineering disciplines | I  M | 2 | Home | General patients | Object | Personal oral care |
| **P14** | **Dental unit for wheelchair users and general patients.** | ^18^ | 2004 | Oral healthcare professionals  Patients and/or public (Patients)  Science and engineering disciplines | I | 2 | Dental practice | Disabled people  General patients | Object | Dental tools and equipment |
| **P15** | ***mVvisualizer*. Easily accessible data exploration for clinicians.** | ^19^ | 2005 | Science and engineering disciplines  Oral healthcare professionals | I | 3 | Dental practice | General patients | Graphical interface | Clinical information systems |
| **P16** | **Human-centred dental discharge summary.** | ^20^ | 2007 | Oral healthcare professionals  Science and engineering disciplines  Patients and/or public (Patients) | I | 3 | Dental practice | General patients | Service | Patient interactions and experience |
| **P17** | **Comparing text-based and graphic user interfaces.** | ^21^ | 2007 | Oral healthcare professionals  Science and engineering disciplines | E | 3 | Dental practice | General patients | Graphical interface | Clinical information systems |
| **P18** | **Design and evaluation of 3D models for electronic dental records.** | ^22^ | 2007 | Science and engineering disciplines  Designers and creative disciplines  Oral healthcare professionals | I  E | 3 | Dental practice | General patients | Graphical interface | Clinical information systems |
| **P19** | **Toothbrush packaging** | ^23^ | 2007 | Designers and creative disciplines | I  M | 2 | Home | General patients | Object | Personal oral care |
| **P20** | **Usability evaluation of electronic dental record systems.** | ^24^ | 2008 | Oral healthcare professionals  Science and engineering disciplines | E | 3 | Dental practice | General patients | Graphical interface | Clinical information systems |
| **P21** | **Dumfries dental centre.** | ^25^ | 2008 | Designers and creative disciplines | I | 2 | Dental practice | General patients | Room or space | Dental office design |
| **P22** | **Improvement of dentists’ gown.** | ^26^ | 2009 | Designers and creative disciplines  Oral healthcare professionals | I  E | 2 | Dental practice | General patients | Apparel | Dental tools and equipment |
| **P23** | ***Teggy*. Mouth brush for nurses brushing patients' teeth.** | ^27^ | 2010 | Designers and creative disciplines  Healthcare professionals | I | 2 | Hospital | Disabled people | Object | Personal oral care |
| **P24** | **Paediatric dental drill for reduced dental anxiety.** | ^28^ | 2010 | Designers and creative disciplines  Patients and/or public (Patients) | I  E | 2 | Dental practice | Children  People with dental anxiety | Object | Dental tools and equipment |
| **P25** | **The humanisation of dental facilities for children.** | ^29^ | 2010 | Designers and creative disciplines | T | 2 | Dental practice | Children  People with dental anxiety | Room or space | Dental office design |
| **P26** | **Ergonomic design for dental offices** | ^30^ | 2010 | Designers and creative disciplines  Healthcare professionals | M  T | 3 | Dental practice | General patients | Process | Clinician health and productivity |
| **P27** | **CD ROM based interactive learning environment for children with dental anxiety.** | ^31^ | 2010 | Science and engineering disciplines  Oral healthcare professionals  Patients and/or public (Patients) | I  E  T | 3 | Home | Children  People with dental anxiety | Graphical interface | Oral health promotion and prevention |
| **P28** | **Dental health game with tangible interface.** | ^32^ | 2010 | Science and engineering disciplines  Patients and/or public (Patients) | I | 3 | Home | Children | Tangible interface | Oral health promotion and prevention |
| **P29** | **Investigation of general dentist's informational needs.** | ^33^ | 2010 | Science and engineering disciplines  Oral healthcare professionals | E | 3 | Dental practice | General patients | Graphical interface | Clinical information systems |
| **P30** | ***HapTEL.* Virtual dental training system.** | ^34^ | 2010 | Science and engineering disciplines  Oral healthcare professionals | I | 3 | Dental school | General patients | Virtual reality | Oral care training |
| **P31** | **Designing a dental clinic for children.** | ^35^ | 2010 | Oral healthcare professionals | M | 2 | Dental practice | Children | Room or space | Dental office design |
| **P32** | **Animation for childrens’ oral health education.** | ^36^ | 2011 | Oral healthcare professionals  Patients and/or public (Patients) | E | 1 | School | Children | Digital material | Oral health literacy |
| **P33** | **Design requirements for dental therapists’ chairs.** | ^37^ | 2011 | Designers and creative disciplines  Oral healthcare professionals | E | 2 | Dental practice | General patients | Object | Dental tools and equipment |
| **P34** | **Influence of positive distractions on children in waiting areas.** | ^38^ | 2011 | Designers and creative disciplines  Patients and/or public (Patients) | E | 3 | Dental practice | Children | Graphical interface | Patient interactions and experience |
| **P35** | **Ergonomically designed dentists chair.** | ^39^ | 2012 | Science and engineering disciplines  Oral healthcare professionals | I  E | 2 | Dental practice | General patients | Object | Dental tools and equipment |
| **P36** | **Surgical tool vending machine.** | ^40^ | 2012 | Science and engineering disciplines | I | 2 | Dental practice | Implant patients | Object | Dental tools and equipment |
| **P37** | **Dental chair headrest redesign.** | ^41^ | 2012 | Science and engineering disciplines | I  M | 2 | Dental practice | General patients | Object | Dental tools and equipment |
| **P38** | **Designing work processes considering tools, seating, customisable environment.** | ^42^ | 2012 | Designers and creative disciplines  Oral healthcare professionals | I  M | 3 | Dental practice | General patients | Process | Clinician health and productivity |
| **P39** | **Method for evaluating and quantifying ergonomic satisfaction level of dental clinics.** | ^43^ | 2012 | Designers and creative disciplines  Oral healthcare professionals | M | 3 | Dental practice | General patients | Process | Clinician health and productivity |
| **P40** | **Investigation of cognitive process and information management for electronic dental record interface design.** | ^44–46^ | 2012 | Oral healthcare professionals  Science and engineering disciplines | E | 3 | Dental practice | General patients | Graphical interface | Clinical information systems |
| **P41** | **Prevention service model for low-income communities.** | ^47^ | 2012 | Designers and creative disciplines  Oral healthcare professionals | I | 3 | No setting/not specified | Socioeconomically disadvantaged people | Service | Oral health promotion and prevention |
| **P42** | **Stories at the Dentist: Dental communication app for patients with intellectual disabilities.** | ^48,49^ | 2013 | Science and engineering disciplines  Oral healthcare professionals  Patients and/or public (Patients) | I | 3 | Dental practice | Disabled people | Graphical interface | Patient interactions and experience |
| **P43** | **Usability of electronic dental records.** | ^50^ | 2013 | Oral healthcare professionals  Science and engineering disciplines | E | 3 | Dental practice | General patients | Graphical interface | Clinical information systems |
| **P44** | **Usability of electronic dental records structured data entry interfaces.** | ^51^ | 2013 | Oral healthcare professionals  Healthcare professionals  Science and engineering disciplines | E | 3 | Dental practice | General patients | Graphical interface | Clinical information systems |
| **P45** | **Virtual reality for pain and discomfort distraction.** | ^52^ | 2013 | Designers and creative disciplines  Oral healthcare professionals  Science and engineering disciplines | I | 3 | Dental practice | Children  People with dental anxiety | Virtual reality | Patient interactions and experience |
| **P46** | ***DAYA.* Children’s toothbrushing game.** | ^53^ | 2014 | Science and engineering disciplines  Patients and/or public (Patients) | I | 3 | Home | Children | 'Smart devices' | Personal oral care |
| **P47** | **Oral health program for Australian Aboriginal children.** | ^54,55^ | 2014 | Oral healthcare professionals  Patients and/or public (Patients and Public) | I  M | 3 | School | Children | Service | Oral health promotion and prevention |
| **P48** | **Design requirements for repurposing dental virtual patients.** | ^56^ | 2014 | Science and engineering disciplines  Oral healthcare professionals | E | 3 | Dental school | General patients | Graphical interface | Oral care training |
| **P49** | **Virtual dental implant training tool.** | ^57^ | 2014 | Science and engineering disciplines | I | 3 | Dental school | Implant patients | Virtual reality | Oral care training |
| **P50** | **Using experience-based design to improve orthodontic care.** | ^58^ | 2014 | Healthcare professionals  Oral healthcare professionals  Patients and/or public (Patients) | M  I | 3 | Hospital | Orthodontics and orthognathic surgery patients | Service | Patient interactions and experience |
| **P51** | **A guide to circulation, layout and design for user experience in dental surgeries.** | ^59^ | 2014 | Designers and creative disciplines | M | 2 | Dental practice | General patients | Room or space | Dental office design |
| **P52** | **Handheld OCT scanning probe.** | ^60^ | 2014 | Oral healthcare professionals  Science and engineering disciplines | I  E | 2 | Dental practice | General patients | Object | Dental tools and equipment |
| **P53** | ***Design Specific.* Dental chairs for special care patients.** | ^61^ | 2015 | Science and engineering disciplines | I | 2 | Dental practice | Disabled people  Bariatric patients | Object | Dental tools and equipment |
| **P54** | **Computer-aided tool for design of ergonomic paediatric dental chairs.** | ^62^ | 2015 | Designers and creative disciplines | I  M | 2 | Dental practice | Children | Object | Dental tools and equipment |
| **P55** | ***Sur-Face.* App for educating patients regarding corrective surgery of facial deformities.** | ^63^ | 2015 | Designers and creative disciplines  Oral healthcare professionals  Patients and/or public (Public) | I  E | 3 | Home | Orthodontics and orthognathic surgery patients | Graphical interface | Informed consent |
| **P56** | **Applying HCI principles in designing usable systems for dentistry** | ^64^ | 2015 | Oral healthcare professionals  Science and engineering disciplines | I  M  T | 3 | Dental practice | General patients | Graphical interface | Clinical information systems |
| **P57** | **Health architecture model and service blueprint of community dental health services.** | ^65^ | 2015 | Designers and creative disciplines  Oral healthcare professionals | M | 4 | No setting/not specified | Community dental service users | Policy | Patient interactions and experience |
| **P58** | ***Hooked on oral hygiene.* Door hanger to remind patients of oral hygiene advice.** | ^66^ | 2016 | Oral healthcare professionals  Designers and creative disciplines | I | 1 | Home | General patients | Printed material | Oral health literacy |
| **P59** | **Arm support for prevention of MSDS in dentists.** | ^67^ | 2016 | Science and engineering disciplines  Oral healthcare professionals | I  E | 2 | Dental practice | General patients | Object | Dental tools and equipment |
| **P60** | **Smart brushing system and oral health database.** | ^68^ | 2016 | Designers and creative disciplines  Patients and/or public (Patients) | I | 3 | Home | General patients | 'Smart devices' | Personal oral care |
| **P61** | ***DenTeach.* Children’s toothbrushing game.** | ^69^ | 2016 | Science and engineering disciplines  Patients and/or public (Patients) | I | 3 | Home | Children | 'Smart devices' | Personal oral care |
| **P62** | **Virtual ergonomic analysis and redesign of a dentist's work cycle.** | ^70^ | 2016 | Designers and creative disciplines | I  E | 3 | Dental practice | General patients | Process | Clinician health and productivity |
| **P63** | **App for patient education in implant dentistry.** | ^71^ | 2016 | Science and engineering disciplines | I  E | 3 | Dental practice | Implant patients | Graphical interface | Informed consent |
| **P64** | ***Chatterbox.* Improving support for socioeconomically disadvantaged families.** | ^72^ | 2016 | Oral healthcare professionals  Designers and creative disciplines  Patients and/or public (Patients) | I | 3 | Home | Socioeconomically disadvantaged people | Service | Oral health promotion and prevention |
| **P65** | **Scientific poster for dental nurse training.** | ^73^ | 2017 | Designers and creative disciplines  Oral healthcare professionals | I | 1 | Dental school | General patients | Printed material | Oral care training |
| **P66** | **Ergonomic evaluation and customized design of toothbrush handle.** | ^74^ | 2017 | Designers and creative disciplines  Patients and/or public (Patients) | E | 2 | Home | General patients | Object | Personal oral care |
| **P67** | **Dental implant positioning system.** | ^75^ | 2017 | Designers and creative disciplines | I  M | 2 | Dental practice | Implant patients | Object | Dental tools and equipment |
| **P68** | **Low-cost dental chair for rural India.** | ^76^ | 2017 | Designers and creative disciplines  Oral healthcare professionals | I  M | 2 | Dental practice | General patients | Object | Dental tools and equipment |
| **P69** | ***Benjamin Brush.* Smart children's toothbrush.** | ^77^ | 2017 | Designers and creative disciplines  Science and engineering disciplines | I | 3 | Home | Children | 'Smart devices' | Personal oral care |
| **P70** | **Inspection of dental photography** | ^78^ | 2017 | Designers and creative disciplines  Oral healthcare professionals | E | 3 | Dental practice | Orthodontics and orthognathic surgery patients | Process | Clinician health and productivity |
| **P71** | **Oral health training in care homes.** | ^79^ | 2017 | Oral healthcare professionals | I  E  M | 3 | Care home | Care home residents |  | Oral care training |
| **P72** | ***Brush Monster.* Smart children's toothbrush.** | ^80^ | 2018 | Designers and creative disciplines | I | 3 | Home | Children | 'Smart devices' | Personal oral care |
| **P73** | ***My Dental.* App for finding local dental clinics, booking appointments, and reminders about upcoming appointments.** | ^81^ | 2018 | Designers and creative disciplines | I | 3 | Home | General patients | Graphical interface | Patient interactions and experience |
| **P74** | **Virtual reality training tool for orthognathic surgery.** | ^82^ | 2018 | Designers and creative disciplines  Science and engineering disciplines  Oral healthcare professionals  Healthcare professionals  Humanities and social science disciplines | I  E | 3 | Dental school | Orthodontics and orthognathic surgery patients | Virtual reality | Oral care training |
| **P75** | **Haptic communication between dentist and patient during treatment.** | ^83^ | 2018 | Designers and creative disciplines  Science and engineering disciplines  Oral healthcare professionals  Patients and/or public (Patients) | I | 3 | Dental practice | People with dental anxiety | Tangible interface | Patient interactions and experience |
| **P76** | ***Jumpstart Mirror Trainer. D*evice for beginner dental students to practice indirect motor skills.** | ^84^ | 2019 | Oral healthcare professionals  Science and engineering disciplines | I  E | 2 | Home | General patients | Object | Oral care training |
| **P77** | ***A-dec 500.* Dental chair.** | ^85^ | 2019 | Science and engineering disciplines  Designers and creative disciplines | I | 2 | Dental practice | General patients | Object | Dental tools and equipment |
| **P78** | **Sleep apnoea appliance.** | ^86^ | 2019 | Oral healthcare professionals  Science and engineering disciplines  Designers and creative disciplines | I | 2 | Home  Dental practice | Sleep apnoea patients | Object | Dental Appliance |
| **P79** | **App aiding dental co-assistants in Indonesia find patients.** | ^87^ | 2019 | Designers and creative disciplines | I | 3 | Home | General patients | Graphical interface | Oral care training |
| **P80** | **Oral health education app for preschoolers.** | ^88^ | 2019 | Oral healthcare professionals  Science and engineering disciplines  Patients and/or public (Patients) | I  E | 3 | Home | Children | Graphical interface | Oral health promotion and prevention |
| **P81** | ***Open Wide and Step Inside*. School oral health program.** | ^89^ | 2019 | Oral healthcare professionals  Designers and creative disciplines  Patients and/or public (Patients & Public) | I  E | 3 | School | Children | Service | Oral health promotion and prevention |
| **P82** | **Oral health program for homeless people.** | ^90,91^ | 2019 | Oral healthcare professionals  Patients and/or public (Patients & Public) | I  E  M | 3 | No setting/not specified | People experiencing homelessness | Service | Oral health promotion and prevention |
| **P83** | **Interface redesign of dental clinic ERP system using design thinking.** | ^92^ | 2019 | Designers and creative disciplines  Oral healthcare professionals | I  M | 3 | Dental practice | General patients | Graphical interface | Clinical information systems |
| **P84** | **Community dental services software.** | ^93^ | 2019 | Science and engineering disciplines  Oral healthcare professionals | I | 3 | Community oral care setting | Community dental service users | Graphical interface | Clinical information systems |
| **P85** | **Touchless interaction with medical images.** | ^94^ | 2019 | Science and engineering disciplines  Oral healthcare professionals | I  E | 3 | Dental practice | General patients | Tangible interface | Clinical information systems |
| **P86** | **Haptic-Audio-Visual Tele-Oral healthcare training simulation.** | ^95^ | 2019 | Science and engineering disciplines | I | 3 | Dental school | General patients | Virtual reality | Oral care training |
| **P87** | ***Whole Mouth Health.* Co-designing oral health literacy resources.** | ^96^ | 2019 | Oral healthcare professionals  Designers and creative disciplines  Science and engineering disciplines  Patients and/or public (Patients & Public)  Humanities and social science disciplines | I  M | 3 | No setting/not specified | General patients | Service | Oral health literacy |
| **P88** | **Patient educational materials for dry mouth.** | ^97^ | 2020 | Designers and creative disciplines  Healthcare professionals  Oral healthcare professionals | I  M | 1 | Pharmacy | General patients | Printed material | Oral health literacy |
| **P89** | **Co-design of an oral health promotion animation for children.** | ^98^ | 2020 | Oral healthcare professionals  Healthcare professionals  Designers and creative disciplines  Patients and/or public (Patients) | I  E  M | 1 | Community hub | Children | Digital material | Oral health literacy |
| **P90** | **Oral care simulator to train nurses brushing disabled patients' teeth.** | ^99,100^ | 2020 | Science and engineering disciplines  Designers and creative disciplines | I | 2 | Hospital  Care home | Disabled people | Object | Oral care training |
| **P91** | ***Todoo.* Automated oral hygiene device.** | ^101^ | 2020 | Designers and creative disciplines | I | 2 | Home | General patients | Object | Personal oral care |
| **P92** | ***Hydrosonic Pro.* Toothbrush.** | ^102^ | 2020 | Designers and creative disciplines | I | 2 | Home | General patients | Object | Personal oral care |
| **P93** | **Modified dental chair for wheelchair users and general patients.** | ^103^ | 2020 | Oral healthcare professionals | I | 2 | Dental practice | Disabled people  General patients | Object | Dental tools and equipment |
| **P94** | **Customized orthodontic appliance using a digital interactive workflow.** | ^104–106^ | 2020 | Designers and creative disciplines | I  M | 3 | Home  Dental practice | Orthodontics and orthognathic surgery patients | Process | Dental Appliance |
| **P95** | **Sustainable brushing system.** | ^107^ | 2020 | Designers and creative disciplines | I | 3 | Home | General patients | 'Smart devices' | Personal oral care |
| **P96** | **Children’s toothbrushing game.** | ^108^ | 2020 | Designers and creative disciplines  Patients and/or public (Patients) | I  E | 3 | Home | Children | 'Smart devices' | Personal oral care |
| **P97** | ***iGAM.* App for monitoring of dental selfies.** | ^109^ | 2020 | Oral healthcare professionals  Science and engineering disciplines  Patients and/or public (Patients) | I | 3 | Home | General patients | Graphical interface | Patient interactions and experience |
| **P98** | **Educational app to reduce infant dental anxiety.** | ^110^ | 2020 | Oral healthcare professionals  Science and engineering disciplines  Patients and/or public (Patients) | I  E | 3 | Home | Children  People with dental anxiety | Graphical interface | Oral health promotion and prevention |
| **P99** | **Patient decision aid for treating obstructive sleep apnoea.** | ^111^ | 2020 | Designers and creative disciplines  Healthcare professionals  Patients and/or public (Patients) | I | 3 | Home | Sleep apnoea patients | Graphical interface | Informed consent |
| **P100** | **Virtual reality dental anxiety mitigation tool.** | ^112^ | 2020 | Designers and creative disciplines  Patients and/or public (Patients) | E  I | 3 | Home | People with dental anxiety | Virtual reality | Patient interactions and experience |
| **P101** | ***STOP.* Oral care experiences of stroke survivors.** | ^113^ | 2020 | Oral healthcare professionals  Designers and creative disciplines  Healthcare professionals  Patients and/or public (Patients)  Humanities and social science disciplines | E | 3 | Home  Dental practice | Stroke survivors | Service | Patient interactions and experience |
| **P102** | **Improving oral care treatment experience for the middle-aged.** | ^114^ | 2020 | Science and engineering disciplines  Patients and/or public (Patients) | I  M  T | 4 | No setting/not specified | General patients | System | Patient interactions and experience |
| **P103** | ***Mouth Matters.* Oral health program for prisoners.** | ^115^ | 2021 | Oral healthcare professionals  Patients and/or public (Patients) | I  M | 3 | Prison | Prisoners | Service | Oral health promotion and prevention |
| **P104** | **An immersive educational tool for dental implant placement.** | ^116^ | 2021 | Science and engineering disciplines  Oral healthcare professionals | I  E | 3 | Dental school | Implant patients | Virtual reality | Oral care training |
| **P105** | ***TOPIC*. resources to help residential care homes manage the oral health of dependent older people.** | ^117, 118^ | 2021 | Designers and creative disciplines  Oral healthcare professionals  Humanities and social science disciplines  Patients and/or public (Patients) | M  I | 3 | Care home | Care home residents | Service | Oral care training |
| **P106** | ***Alivia.* Tangible communication between patient and dentist** | ^119^ | 2021 | Designers and creative disciplines  Patients and/or public (Patients and Public) | E  M  I | 3 | Dental practice | People with dental anxiety | Tangible interface | Patient interactions and experience |
| **P107** | **Diet and oral health app.** | ^120^ | 2021 | Oral healthcare professionals  Science and engineering disciplines  Humanities and social science disciplines  Patients and/or public (Patients) | I  E | 3 | No setting/not specified | Children | Graphical interface | Oral health promotion and prevention |
| **P108** | **Smartbrush oral health installation for aged care centres.** | ^121^ | 2021 | Healthcare professionals  Science and engineering disciplines  Oral healthcare professionals | E | 3 | Care home | Care home residents | 'Smart devices' | Personal oral care |
| **P109** | **Tool handle parameters for MSDS** | ^122^ | 2021 | Designers and creative disciplines  Oral healthcare professionals | E | 2 | Dental practice | General patients | Object | Dental tools and equipment |
| **P110** | **A serious game for training in child protection.** | ^123^ | 2021 | Oral healthcare professionals  Designers and creative disciplines | E  I  M  T | 3 | Dental school | Children | Service | Oral care training |
| **P111** | **Design game for adolescents to talk about oral health.** | ^124^ | 2022 | Designers and creative disciplines  Patients and/or public (Patients) | I  M | 3 | School | Children | Service | Oral health promotion and prevention |
| **P112** | **Video on silver diammine fluoride for children.** | ^12^ | 2022 | Oral healthcare professionals  Patients and/or public (Patients) | M  I  E | 1 | No setting/not specified | Children | Digital material | Oral health promotion and prevention |
| **P113** | **Operative dentistry virtual reality simulator.** | ^126^ | 2022 | Oral healthcare professionals  Science and engineering disciplines | I  E | 3 | Dental school | General patients | Virtual reality | Oral care training |
| **P114** | ***HABIT.* Oral health intervention delivered by health visitors for parents of children aged 9–12 months.** | ^127^ | 2022 | Oral healthcare professionals  Healthcare professionals  Humanities and social science disciplines | I  M | 3 | Home | Children | Service | Oral health promotion and prevention |
| **P115** | **Smartphone system for management of children's oral health.** | ^128^ | 2022 | Oral healthcare professionals | I  M | 3 | Home | Children | Graphical interface | Oral health promotion and prevention |
| **P116** | **Local anaesthetic dentistry jet injection device.** | ^129^ | 2022 | Science and engineering disciplines  Oral healthcare professionals | I | 2 | Dental practice | General patients | Object | Dental tools and equipment |
| **P117** | ***Oro White.* Toothbrush with dental caries detection.** | ^130^ | 2022 | Science and engineering disciplines | I | 3 | Home | General patients | 'Smart devices' | Personal oral care |
| **P118** | **TENS for dental injection.** | ^131^ | 2022 | Designers and creative disciplines | I | 2 | Dental practice | General patients | Object | Dental tools and equipment |
| **P119** | **Cartoon graphics in dental spaces for children.** | ^132^ | 2022 | Designers and creative disciplines | I  T | 2 | Dental practice | General patients | Room or space | Dental office design |

##

## 3.1. References

1 FDI. FDI’s definition of oral health. 2016.https://www.fdiworlddental.org/fdis-definition-oral-health (accessed 16 Jun2021).

2 Evans TE Jr, Lucaccini LF, Hazell JW, Lucas RJ. Evaluation of Dental Hand Instruments. *Human Factors* 1973; **15**: 401–406.

3 Rommerdale EH, Comer RW, Caughman WF. University of Mississippi Dental Care Unit: toothbrushing for the handicapped. *Special Care in Dentistry* 1983; **3**: 108–109.

4 Baycar R, Aker F, Serowski A, Bailey G. Mobile oral treatment and examination chair. *Special Care in Dentistry* 1983; **3**: 224–225.

5 Baycar R, Aker F, Serowski A. Portable dental chair. *Special Care in Dentistry* 1983; **3**: 57–60.

6 Bill DJ, Weddell JA. Dental office access for patients with disabling conditions. *Special Care in Dentistry* 1986; **6**: 246–252.

7 Schrock JM, Cooper GJ. Dental Office Design: Perceptions of Dentists in Texas. *Journal of Interior Design* 1987; **13**: 47–52.

8 Bruder R. Integrating ergonomic findings into the design of new products for dental. *Global Ergonomics* 1998; : 299.

9 Unthank M, True G. Interior design for dentistry. *The Journal of the American Dental Association* 1999; **130**: 1586–1590.

10 Waterpik Flosser designer wins award. Dentistry IQ. 2001.https://www.dentistryiq.com/practice-management/industry/article/16355755/waterpik-flosser-designer-wins-award (accessed 4 Aug2021).

11 Koch S. Designing clinically useful systems: examples from medicine and dentistry. *Advances in dental research* 2003; **17**: 65–68.

12 Cederman-Haysom T, Brereton M. A participatory design agenda for ubiquitous computing and multimodal interaction: A case study of dental practice. Association for Computing Machinery: Trento, Italy, 2006, pp 11–20.

13 Campbell B, Cederman-Haysom T, Donovan J, Brereton M. Springboards into design: Exploring multiple representations of interaction in a dental surgery. In: *Proc OZCHI*. Brisbane, Australia, 2003, pp 14–23.

14 Campbell B, Brereton M. Maintaining Human Agency in the Design of Context-Aware Systems: Design Games in a Dental Surgery. 2004.

15 Donovan J, Brereton M. Meaning in movement: A gestural design game. 2004, pp 163–166.

16 Johnson WL, Shaw E, Marshall A, LaBore C. Evolution of user interaction: the case of agent adele. 2003, pp 93–100.

17 Hohlbein DJ, Williams M, Mintel T. Driving toothbrush innovation through a cross-functional development team. *Whole Mouth Clean* 2004; **25**: 7.

18 Tamazawa Y, Watanabe M, Kikuchi M *et al.* A new dental unit for both patients in wheelchairs and general patients. *Gerodontology* 2004; **21**: 53–59.

19 Erichson N, Torgersson O. mVisualizer: Easily Accessible Data Exploration for Clinicians. In: Engelbrecht R, Geissbuhler A, Lovis C, Mihalas G (eds). *Connecting Medical Informatics and Bio-Informatics*. Ios Press: Amsterdam, 2005, pp 725–730.

20 Walji M, Loeffelholz J, Valenza JA. A human-centered design of a dental discharge summary (DDS) for patients. *AMIA Annu Symp Proc* 2007; : 1146.

21 Chen J-W, Zhang J. Comparing Text-based and Graphic User Interfaces for novice and expert users. *AMIA . Annual Symposium proceedings AMIA Symposium* 2007; : 125–129.

22 Marotta MF, Phanichphant P, Malatack P *et al.* Design and evaluation of 3d models for electronic dental records. ACM Press, 2007 doi:10.1145/1240866.1241042.

23 DOGA. Jordan toothbrush packaging. https://doga.no/en/tools/inclusive-design/cases/jordan-toothbrush-packaging/ (accessed 19 Nov2021).

24 Thyvalikakath TP, Monaco V, Thambuganipalle HB, Schleyer T. A usability evaluation of four commercial dental computer-based patient record systems. *The Journal of the American Dental Association* 2008; **139**: 1632–1642.

25 Architecture and Design Scotland. Dumfries Dental Centre Case Study: produced by the Healthcare Design Programme. 2008https://www.ads.org.uk/case-study-dumfries-dental-centre/.

26 Schacher M, Haïkel Y, Berger S, Schacher L, Adolphe DC. Improvement of dentist gowns–new constraints and new risks. *International Journal of Clothing Science and Technology* 2009.https://www.emerald.com/insight/content/doi/10.1108/09556220910959963/full/html.

27 Coventry University HDTI. Teggy. 2010.https://www.coventry.ac.uk/business/our-services/health-design-technology-institute/health-design-technology-institute-case-study-menu/teggy/ (accessed 20 Dec3AD).

28 Reynolds A, Liu TL. The Study of Children’s Emotional Responses and Its Application to the Redesign of the Traditional Dental Handpiece. Zenodo, 2019 doi:10.5281/zenodo.2596210.

29 Shi Y, Hu F. The humanization design of dental facilities for children. IEEE, 2010, pp 585–588.

30 Ahearn DJ, Sanders MJ, Turcotte C. Ergonomic design for dental offices. *Work* 2010; **35**: 495–503.

31 Salam SN-A, Yahaya WAJ-W, Ali A-M. Using Persuasive Design Principles in Motivational Feeling towards Children Dental Anxiety (CDA). In: *Persuasive Technology*. Springer Berlin Heidelberg, 2010, pp 223–237.

32 Gerling K, Klauser M, Masuch M. Serious interface design for dental health: Wiimote-based tangible interaction for school children. In: *CEUR Workshop Proceedings*. 2010.

33 Song M, Spallek H, Polk D, Schleyer T, Wali T. How information systems should support the information needs of general dentists in clinical settings: suggestions from a qualitative study. *BMC Medical Informatics and Decision Making* 2010; **10**: 7.

34 Tse B, Harwin W, Barrow A, Quinn B, San Diego J, Cox M. Design and Development of a Haptic Dental Training System - hapTEL. In: *Haptics: Generating and Perceiving Tangible Sensations*. Springer Berlin Heidelberg, 2010, pp 101–108.

35 Ashwin J. Designing a Dental Clinic for Children. 2010, p 12.

36 Sinor MZ. Comparison between conventional health promotion and use of cartoon animation in delivering oral health education. *Intl J humanities and social science* 2011; **1**: 169–174.

37 Domljan D, Grbac I, Vlaović Z, Carek V, Čolić S. Design requirements for working chairs of a dental therapist. 2011, p 33.

38 Pati D, Nanda U. Influence of Positive Distractions on Children in Two Clinic Waiting Areas. *HERD: Health Environments Research & Design Journal* 2011; **4**: 124–140.

39 Haddad O, Sanjari MA, Amirfazli A, Narimani R, Parnianpour M. Trapezius muscle activity in using ordinary and ergonomically designed dentistry chairs. *Int J Occup Environ Med* 2012; **3**: 76–83.

40 Nelson CA, Hossain S, Al-Okaily A, Ong J. A novel vending machine for supplying root canal tools during surgery. *Journal of medical engineering & technology* 2012; **36**: 102–116.

41 Ito T, Ichikawa T, Hanumara NC, Slocum AH. Expectation Management in a Global Collaboration Project Using a Deterministic Design Approach. American Society of Mechanical Engineers, 2012, pp 573–580.

42 Kar G, Mullick A. Designing with Users: A case study for design of dental workspace. SAGE Publications Sage CA: Los Angeles, CA, 2012, pp 652–655.

43 Orenha ES, Naressi SMC, Teixeira SC *et al.* Assessment of Ergonomic Aspects in Dental Clinic Rooms. *Work* 2012; **41**: 6103–6106.

44 Thyvalikakath TP, Dziabiak MP, Johnson R *et al.* Advancing cognitive engineering methods to support user interface design for electronic health records. *International journal of medical informatics* 2014; **83**: 292–302.

45 Thyvalikakath TP, Dziabiak MP, Johnson R, Torres-Urquidy MH, Yabes J, Schleyer TK. Designing Clinical Data Presentation in Electronic Dental Records Using Cognitive Task Analysis Methods. IEEE, 2012 doi:10.1109/hisb.2012.24.

46 Acharya A, Hernandez P, Thyvalikakath T, Ye H, Song M, Schleyer T. Development and initial validation of a content taxonomy for patient records in general dentistry. *Int J Med Inform* 2013; **82**: 1171–82.

47 Attaie AB, Burow L. Changing how we care: innovating the dental care experience for underserved communities in New York City. *Touchpoint* 2015; **3**: 48–53.

48 Menzies R, Herron D, Scott L, Freeman R, Waller A. Involving clinical staff in the design of a support tool to improve dental communication for patients with Intellectual Disabilities. 2013 doi:10.1145/2513383.2513407.

49 Speech App Helps Disabled People to Communicate at the Dentist. https://discovery.dundee.ac.uk/en/clippings/speech-app-helps-disabled-people-to-communicate-at-the-dentist (accessed 20 Dec5AD).

50 Suebnukarn S, Rittipakorn P, Thongyoi B, Boonpitak K, Wongsapai M, Pakdeesan P. Usability assessment of an electronic health record in a comprehensive dental clinic. *SpringerPlus* 2013; **2**: 220.

51 Walji MF, Kalenderian E, Tran D *et al.* Detection and characterization of usability problems in structured data entry interfaces in dentistry. *International Journal of Medical Informatics* 2013; **82**: 128–138.

52 Bidarra R, Gambon D, Kooij R, Nagel D, Schutjes M, Tziouvara I. Gaming at the dentist’s – serious game design for pain and discomfort distraction. In: *Games for Health*. Springer Fachmedien Wiesbaden, 2013, pp 207–215.

53 Shao K, Li R, Huang J, Wu J, Song H. DAYA: A system for monitoring and enhancing children’s oral hygiene. 2014, pp 251–256.

54 Dimitropoulos Y, Gwynne K, Blinkhorn A, Holden A. A school fluoride varnish program for Aboriginal children in rural New South Wales, Australia. *Health Promot J Austr* 2020; **31**: 172–176.

55 Dimitropoulos Y, Holden A, Gwynne K, Do L, Byun R, Sohn W. Outcomes of a co-designed, community-led oral health promotion program for Aboriginal children in rural and remote communities in New South Wales, Australia. *Community Dent Health* 2020; **37**: 132–137.

56 Antoniou PE, Athanasopoulou CA, Dafli E, Bamidis PD. Exploring design requirements for repurposing dental virtual patients from the web to second life: a focus group study. *J Med Internet Res* 2014; **16**: e151.

57 Jeppsen I. Development Methods: Virtual Dental Implant Trainer. *Design and Development of Training Games: Practical Guidelines from a Multidisciplinary Perspective* 2014.

58 Ellis PE, Silverton S. Using the experience-based design approach to improve orthodontic care. *Journal of orthodontics* 2014; **41**: 337–344.

59 Architecture and Design Scotland. Dental Surgery Design: A quick guide illustrating the impact of circulation, layout and design on the user experience. 2014https://www.ads.org.uk/wp-content/uploads/final-copy-for-web.pdf.

60 Demian D, Duma V-F, Sinescu C *et al.* Design and testing of prototype handheld scanning probes for optical coherence tomography. *Proceedings of the Institution of Mechanical Engineers, Part H: Journal of Engineering in Medicine* 2014; **228**: 743–753.

61 Design Specific. https://www.designspecific.co.uk/ (accessed 3 Dec2020).

62 Su Y. The research and design of children’s dental treatment machine based on ergonomics. Proceedings of Science (PoS): Guangzhou, China, 2015.

63 Pulijala Y, Ma M, Ayoub A. Design and Development of Sur-Face: An interactive mobile app for educating patients regarding corrective surgery of facial deformities. In: *Serious Games*. Springer International Publishing, 2015, pp 26–35.

64 Kalenderian E, Walji M, Ramoni R. Applying HCI principles in designing usable systems for dentistry. In: *Cognitive Informatics for Biomedicine*. Springer, 2015, pp 189–213.

65 Lievesley M, Wassall R. Designing across organisational boundaries-Community Dentistry Services. In: *Third European Conference on Design4Health*. Newcastle University, 2015.

66 Coles M. ‘To see my idea come to life has been totally amazing’. *BDJ Team* 2016; **3**: 16121.

67 Hallaj S, Razi SSM. Design and Evaluation of an Arm Support for Prevention of MSDs in Dentists. In: Rebelo F, Soares M (eds). *Advances in Ergonomics in Design*. Springer International Publishing Ag: Cham, 2016, pp 265–275.

68 Nie J. *Oral Hygiene System Design*. 2016.

69 Lee N, Jang D, Kim Y, Bae B-C, Cho J-D. Denteach: A device for fostering children’s good tooth-brushing habits. 2016, pp 619–624.

70 Bruno F, Califano R, Greco R, Ricco G, Vallone M, Valter N. Virtual ergonomic analysis and redesign of a dentist’s workcycle. *ARPN Journal of Engineering and Applied Sciences* 2016; **11**: 12371–12379.

71 Canbazoglu E, Salman YB, Yildirim ME, Merdenyan B, Ince IF. Developing a mobile application to better inform patients and enable effective consultation in implant dentistry. *Computational and structural biotechnology journal* 2016; **14**: 252–261.

72 Nanjappa S, Lindsay R, White H, Freeman R. Chatterbox: A Multidisciplinary Approach to Co-design. In: *11th European Academy of Design Conference*. Paris, France, 2015.

73 Lewis H. Case study – human body relay race. *Journal of Visual Communication in Medicine* 2017; **40**: 66–71.

74 Bhatia V, Bhatia A, Kalra P, Singh J, Datta R. Ergonomic Evaluation and Customized Design of Toothbrush Handle. In: *Research into Design for Communities, Volume 1*. Springer Singapore, 2017, pp 205–217.

75 Chan T-H. Utilize SCAMPER to design a navigation system for dental implant. IEEE, 2017, pp 574–577.

76 Kundal A, Chatterjee J, Roy S. From Design Thinking to Design Doing—A Procedural Approach Creating Socially Responsive Artifacts—A Case of Designing Towards Affordable Dental Care (for India). In: *Research into Design for Communities, Volume 1*. Springer Singapore, 2017, pp 973–986.

77 McLaughlin A. Map’s smart toothbrush looks to makes brushing teeth ‘fun’. Design Week (Online Edition). 2017; : 2–2.

78 Altiparmakogullari Y, Cifter M, Cifter AS. A Multidisciplinary inspection of Dental Photography: What Do Dentist Think and What Can Designer Do? *Des J* 2017; **20**: S1989–S1997.

79 Patel R, Robertson C, Gallagher JE. Collaborating for oral health in support of vulnerable older people: co-production of oral health training in care homes. *Journal of Public Health* 2017; **41**: 164–169.

80 Dawood S. Brush Monster uses AR game to make brushing teeth “fun and engaging”. 2018.https://www.designweek.co.uk/issues/14-20-may-2018/brush-monster-uses-ar-game-make-brushing-teeth-fun-engaging/#:~:text=A%20smart%20toothbrush%20kit%20has,and%20apps%20aimed%20at%20children.

81 Patel S. UI/UX Casestudy – My Dental iOS App. 2018.https://shremal.com/portfolios/ui-ux-casestudy-my-dental-ios-app (accessed 20 Dec3AD).

82 Pulijala Y, Ma M, Pears M, Peebles D, Ayoub A. An innovative virtual reality training tool for orthognathic surgery. *International Journal of Oral and Maxillofacial Surgery* 2018; **47**: 1199–1205.

83 Guribye F, Gjøsæter T. Tangible Interaction in the Dentist Office. ACM, 2018 doi:10.1145/3173225.3173287.

84 McClure AR, Roomian TC, Eisen SE, Kugel G, Amato RB. Jumpstart Mirror Trainer: a New Device for Teaching Mirror Skills to First-Year Dental Students. *Journal of dental education* 2019; **83**: 1199‐1204.

85 The new A-dec 500: a legend, redefined. *British Dental Journal* 2019; **226**: 906–906.

86 García NM, Blaya F, Urquijo EL, Heras ES, D’Amato R. Oral appliance for Obstructive Sleep Apnea: Prototyping and Optimization of the Mandibular Protrusion Device. *Journal of medical systems* 2019; **43**: 107.

87 Widhiansyah R. UX Case Study: Designing a Mobile App for Dental Co-Assistant in Indonesia. https://medium.com/@widhiandraw/ux-case-study-designing-a-mobile-app-for-dental-co-assistant-in-indonesia-c620d3893fab (accessed 20 Dec3AD).

88 Campos LFXA, Cavalcante JP, Machado DP, Marçal E, Silva PGDB, Rolim JPML. Development and Evaluation of a Mobile Oral Health Application for Preschoolers. *Telemedicine and e-Health* 2019; **25**: 492–498.

89 Witton R, Smith W. A service evaluation of the ‘Open Wide and Step Inside’ school oral health programme. *BDJ Open* 2019; **5**: 6.

90 Rodriguez A, Beaton L, Freeman R. Strengthening Social Interactions and Constructing New Oral Health and Health Knowledge: The Co-design, Implementation and Evaluation of A Pedagogical Workshop Program with and for Homeless Young People. *Dentistry Journal* 2019; **7**: 11.

91 Dickson K, Rodriguez A, Freeman R, Gupta E, Walkden C. A qualitative study of the Scottish homeless service provisions through the production of reflexive mapping exercises. *The Lancet* 2021; **398**: S41.

92 Suzianti A, Arrafah G. User Interface Redesign of Dental Clinic ERP System using Design Thinking. ACM Press, 2019 doi:10.1145/3364335.3364369.

93 Valentine L, Wassall R. Co-designing Community Dental Services software. 2019, pp 1735-1743-1735–1743.

94 Paulo SF, Relvas F, Nicolau H *et al.* Touchless interaction with medical images based on 3D hand cursors supported by single-foot input: A case study in dentistry. *Journal of Biomedical Informatics* 2019; **100**: 103316.

95 Iiyoshi K, Tauseef M, Gebremedhin R *et al.* Final Report Design of a Haptic-Audio-Visual Tele-Dental Training Simulation. 2019.

96 FDI Whole Mouth Health. Lab4Living | Design & Health or Well-being | Sheffield Hallam University. https://lab4living.org.uk/projects/whole-mouth-health/ (accessed 13 Sep2021).

97 Juntos. Patient education materials – health promotion campaign. 2020.https://www.juntosmarketing.com.au/case-studies/oral-health-campaign-materials/ (accessed 3 Dec2020).

98 Scott DAH, Currie C, Stones T, Scott C, John J, Wanyonyi K. Co-design of an oral health promotion animated film with families in the South of England. *Br Dent J* 2020; **228**: 164–170.

99 Daigo T, Muramatsu M, Mitani A. Development of the second prototype of an oral care simulator. *Journal of Robotics and Mechatronics* 2021; **33**: 172–179.

100 Herath B, Dewmin G, Sukumaran S *et al.* Design and Development of a Novel Oral Care Simulator for the Training of Nurses. *IEEE Transactions on Biomedical Engineering* 2020; **67**: 1314–1320.

101 A’ Design Award & Competition. Toodo Oral Hygiene Cleaning Device by Tairan Li and Chao Huang. 2020.https://competition.adesignaward.com/design.php?ID=93244 (accessed 4 Aug2021).

102 Erdmann Design AG. Erdmann Design toothbrush solution wins German Design Award for Curaprox. 2020.https://www.pharmaceutical-networking.com/erdmann-design-toothbrush-solution-wins-german-design-award-for-curaprox/.

103 Lakshmi K, Madankumar PD. Development of modified dental chair to accomodate both wheelchair bound patients and general population. *Disability and Rehabilitation: Assistive Technology* 2020; **15**: 467–470.

104 Filippi S, Grigolato L, Savio G. UX Concerns in Developing Functional Orthodontic Appliances. Springer, 2020, pp 229–241.

105 Grigolato L, Filippi S, Barattin D *et al.* Conceptual Design of a Functional Orthodontic Appliance for the Correction of Skeletal Class II Malocclusion. In: *Lecture Notes in Mechanical Engineering*. Springer International Publishing, 2020, pp 329–341.

106 Grigolato L, Filippi S, Cantarella D *et al.* Concept selection and interactive design of an orthodontic functional appliance. *International Journal on Interactive Design and Manufacturing (IJIDeM)* 2020. doi:10.1007/s12008-020-00743-z.

107 Sustainable Dental Routines / Dental Cleaning. FH JOANNEUM. https://www.fh-joanneum.at/en/project/sustainable-dental-routines-dental-cleaning/ (accessed 16 Feb2023).

108 Chuko C, Chao F-L, Tsai H-Y. Design of interactive aids for children’s teeth cleaning habits. *Advances in Science, Technology and Engineering Systems Journal* 2020; **5**: 494–499.

109 Tobias G, Spanier AB. Developing a Mobile App (iGAM) to Promote Gingival Health by Professional Monitoring of Dental Selfies: User-Centered Design Approach. *JMIR mHealth and uHealth* 2020; **8**: e19433.

110 Coutinho MB, Damasceno JX, Mesquita Cals de Oliveira PC, Alves Marinho IM, Marçal E, Gomes Fernandes Vieira-Meyer AP. A Novel Mobile App Intervention to Reduce Dental Anxiety in Infant Patients. *Telemedicine and e-Health* 2020; **27**: 694–700.

111 Lo H-C, Yang M-C, Lin F-N. A Prototype of Patient Decision Aid for Treating Obstructive Sleep Apnea. In: *Human Aspects of IT for the Aged Population. Healthy and Active Aging*. Springer International Publishing, 2020, pp 128–137.

112 Han T, Xiao H, Shen T, Xie Y, Zhu Z. A Virtual Reality Dental Anxiety Mitigation Tool Based on Computerized Cognitive Behavioral Therapy. In: Chen JYC, Fragomeni G (eds). . Springer International Publishing: Cham, 2020, pp 385–398.

113 O’Malley L, Powell R, Hulme S *et al.* A qualitative exploration of oral health care among stroke survivors living in the community. *Health Expectations* 2020; **23**: 1086–1095.

114 Chen S, Li Y. A Study on Oral Health Care System Designing for the Middle-Aged Based on SAPAD-PCA. In: *International Conference on Human-Computer Interaction*. Springer, 2020, pp 639–653.

115 Freeman R. Oral Health as a Door to Promoting Psychosocial Functioning for People in Custody: Lessons Learnt from the Development of the Mouth Matters Intervention. In: *Issues and Innovations in Prison Health Research*. Springer International Publishing, 2021, pp 211–233.

116 Zorzal ER, Paulo SF, Rodrigues P, Mendes JJ, Lopes DS. An immersive educational tool for dental implant placement: A study on user acceptance. *International Journal of Medical Informatics* 2021; **146**: 104342.

117 Improving the Oral Health of Older People in Care Homes: a Feasibility Study (TOPIC) - NIHR Funding and Awards. https://dev.fundingawards.nihr.ac.uk/award/17/03/11 (accessed 9 May2022).

118 Langley J, Wassall R, Geddis-Regan A *et al.* Putting guidelines into practice: Using co-design to develop a complex intervention based on NG48 to enable care staff to provide daily oral care to older people living in care homes. *Gerodontology* 2022; **40**: 112–126.

119 Gutiérrez C, Barteld C. *Alivia. Communication between patient and dentist.* 2021.https://industrialdesign.zhdk.ch/site/assets/files/4857/dokumentation_alivia.pdf (accessed 17 Feb2022).

120 Akmal Muhamat N, Hasan R, Saddki N, Mohd Arshad MR, Ahmad M. Development and usability testing of mobile application on diet and oral health. *PLoS One* 2021; **16**: e0257035.

121 Grzegorz Broda L, Oseni T, Stranieri A, Marino R, Robinson J, Yates M. The Design of a Smartbrush Oral Health Installation for Aged Care Centres in Australia. 2021, pp 176–180.

122 Bhatia V, Randhawa JS, Kalra P, Jain A, Grover V. Effect of Tool Handle Design Parameters on Upper Extremity Muscle Performance in Periodontology. Springer, 2021, pp 25–37.

123 Park C. An investigation of the barriers to referral in child protection for dental team professionals through serious game design. 2021.

124 Jasbi A. *Design Game for co-design workshops with the scope of improving dental health for adolescents*. 2022.https://ntnuopen.ntnu.no/ntnu-xmlui/handle/11250/2988951 (accessed 17 Feb2023).

125 Timms L, Marshman Z, Deery C, Rodd H. Co-production of an information video on silver diammine fluoride–with children, for children. *Faculty Dental Journal* 2022; **13**: 160–165.

126 Rodrigues P, Esteves A, Botelho J *et al.* Usability, acceptance, and educational usefulness study of a new haptic operative dentistry virtual reality simulator. *Computer Methods and Programs in Biomedicine* 2022; **221**: 106831.

127 Owen J, Gray-Burrows KA, Eskytė I *et al.* Co-design of an oral health intervention (HABIT) delivered by health visitors for parents of children aged 9–12 months. *BMC Public Health* 2022; **22**: 1–14.

128 Zhang C, Ran L, Chai Z, Yu C, Song J. The design, development and usability testing of a smartphone-based mobile system for management of children’s oral health. *Health Informatics Journal* 2022; **28**: 14604582221113432.

129 Weatherly K, Brunton P, Loch C *et al.* Case study of user experience-driven design in a new local anaesthetic dentistry jet injection device. *Journal of the Royal Society of New Zealand* 2022; : 1–13.

130 Kalita B, Sharma A. Oro White Toothbrush| Design and Conceptualizing Dental Caries Detection Method. In: *Ergonomics for Design and Innovation: Humanizing Work and Work Environment: Proceedings of HWWE 2021*. Springer, 2022, pp 1189–1199.

131 Xu A. TENS for dental injection. Product Design Engineering Degree Show. 2022.https://www.pdedegreeshow.com/aowen-xu (accessed 17 Feb2023).

132 Zhu L, Wei Z. Research on the application of computer graphics aided design in cartoon graphics design of children’s hospital. IEEE, 2022, pp 917–920.

#

# 4. Appendix E: Visual Mapping Process

Figure 1 outlines the visual mapping process. Figures 2-7 shows iterations of the visualisations presented in chronological order. The mapping process was conducted in parallel to data extraction and coding. Versions of the map, including themes, relationships and visuals were discussed regularly by the research team. As a result, the content of the maps in figures 1-6 evolve as more projects were added, and as themes were identified, redefined and refined.

*Figure 1. Visual Mapping Process.*


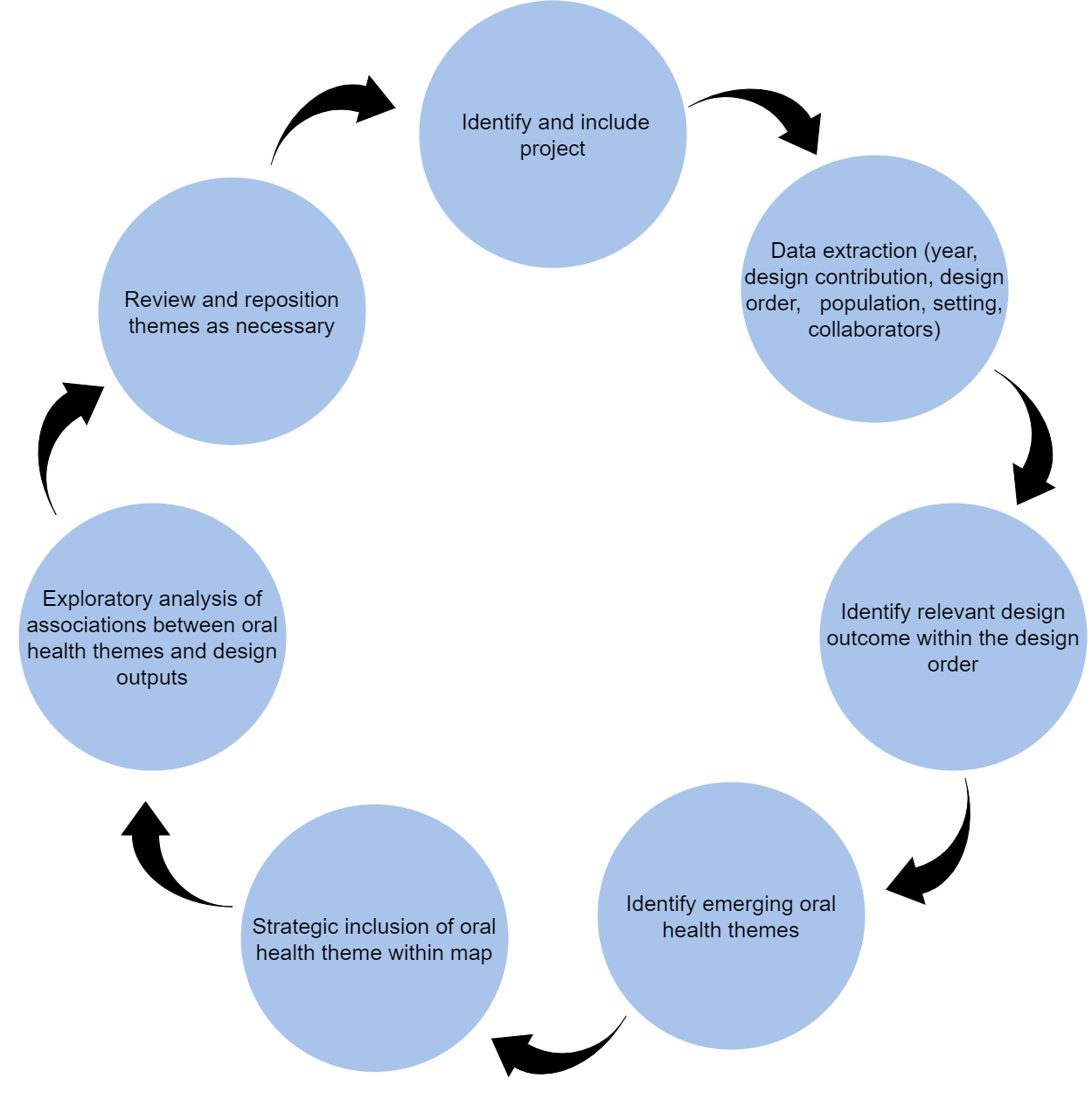


*Figure 2. Early mapping exploration. Some initial themes and visualisation.*

*
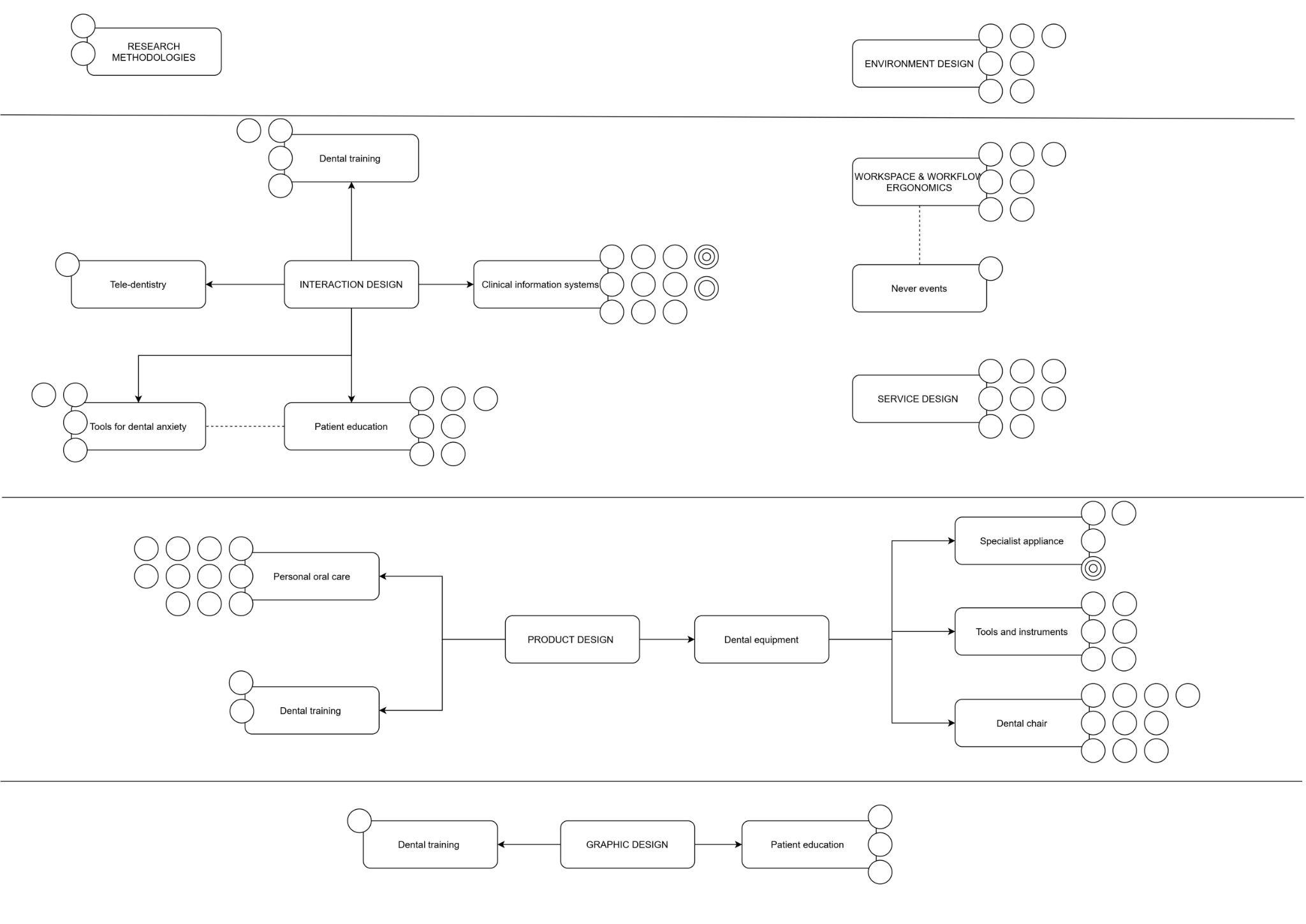
*

*Figure 3. Early mapping exploration. Relationship between potential themes and stakeholders.*

*
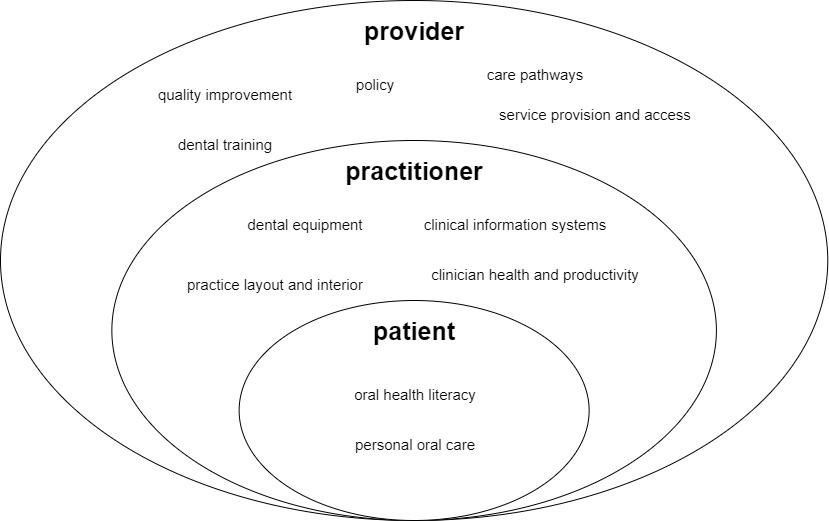
*

*Figure 4. Developing map layout, legend, themes.*


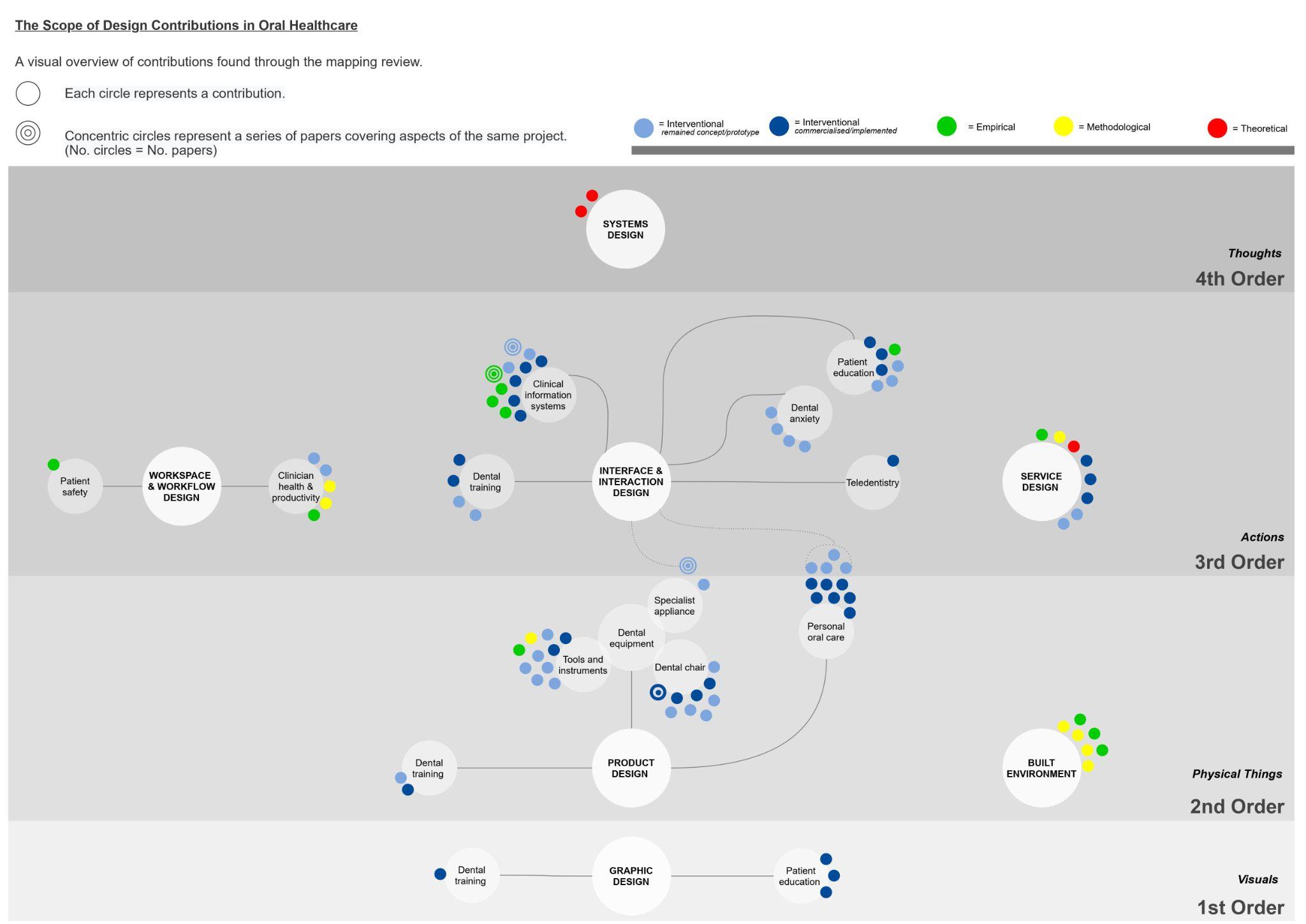


*Figure 5. Making the map interactive.*


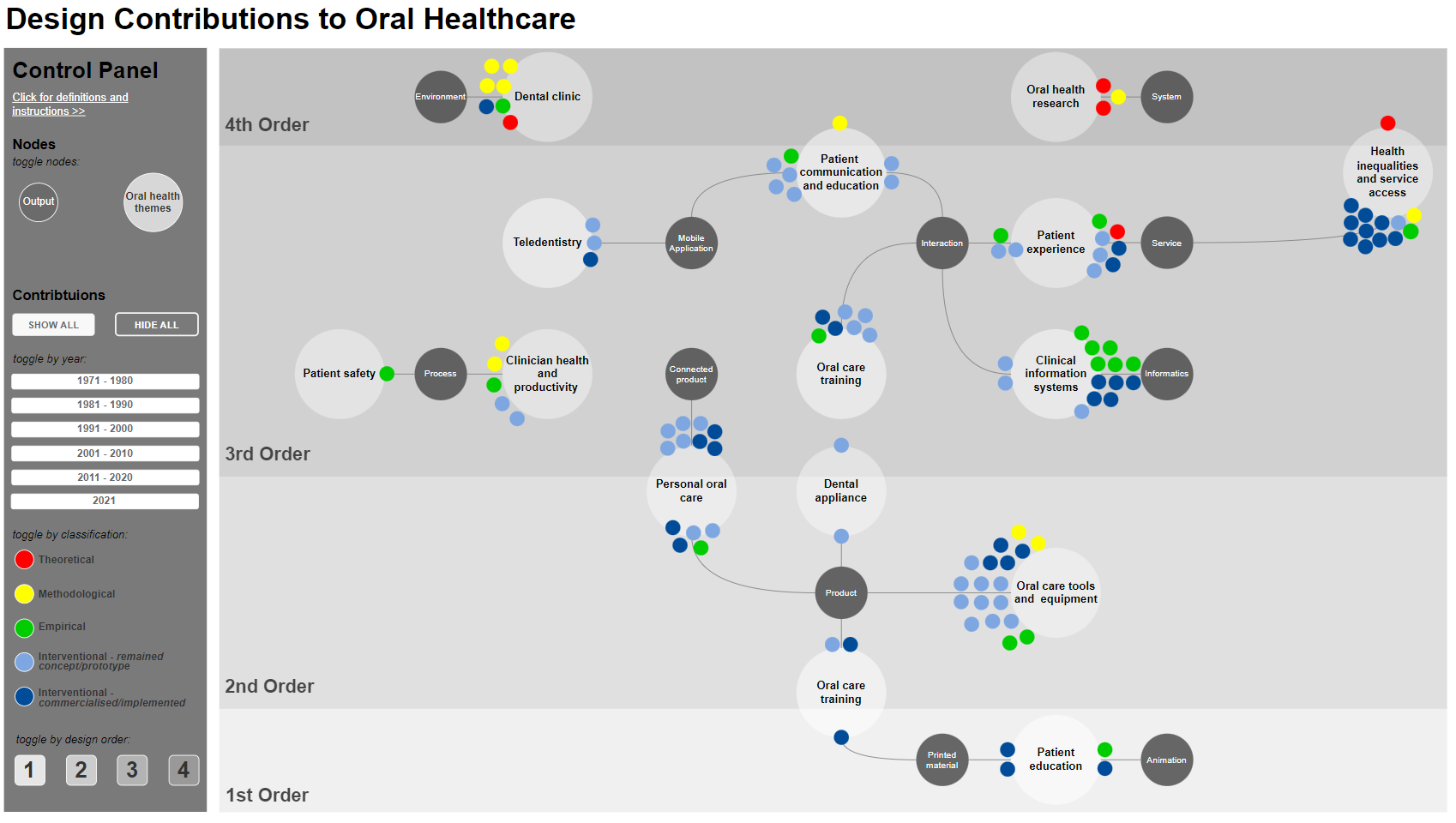


*Figure 6. Updating projects and further theme exploration.*


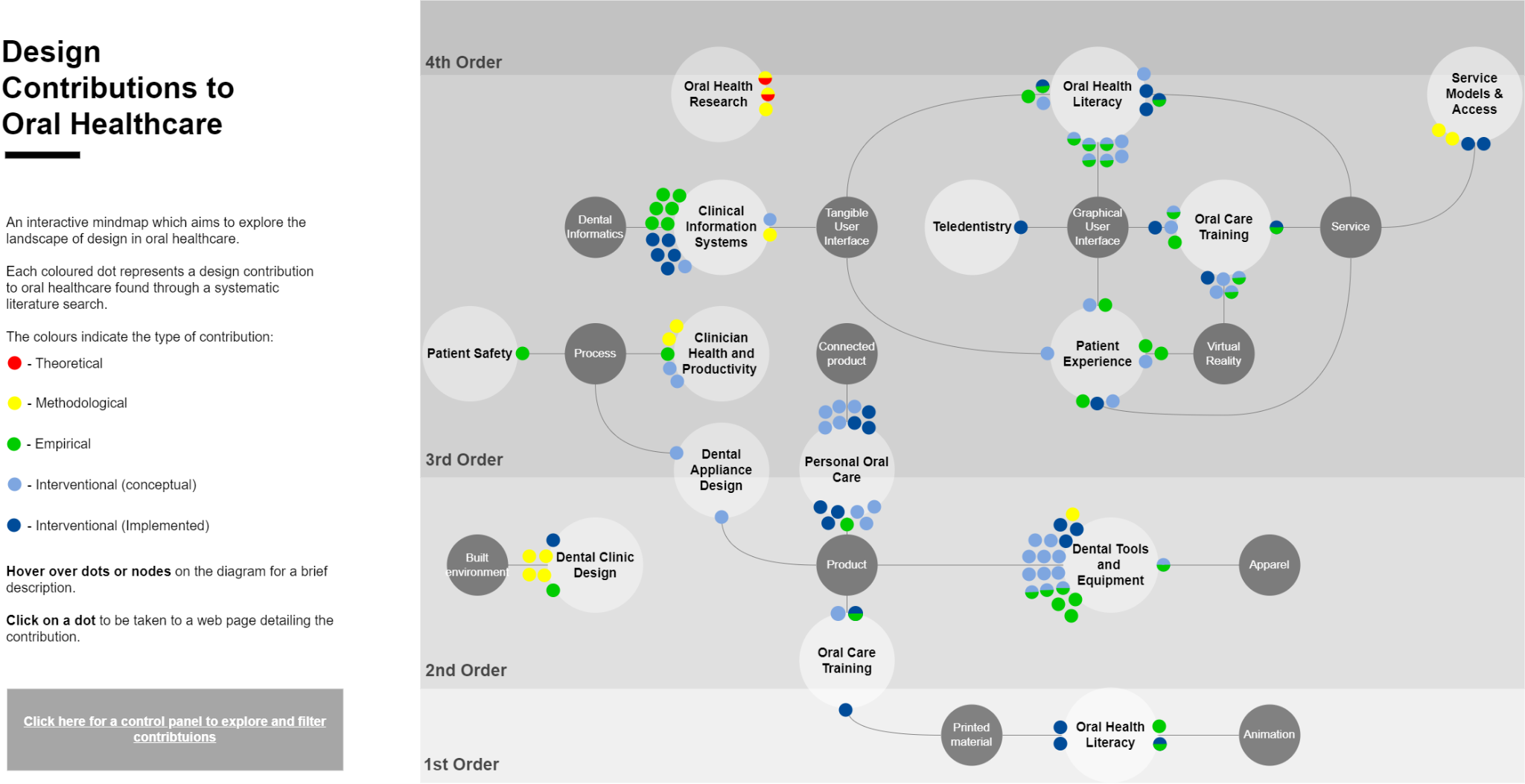


*Figure 7. Latest interactive map.*


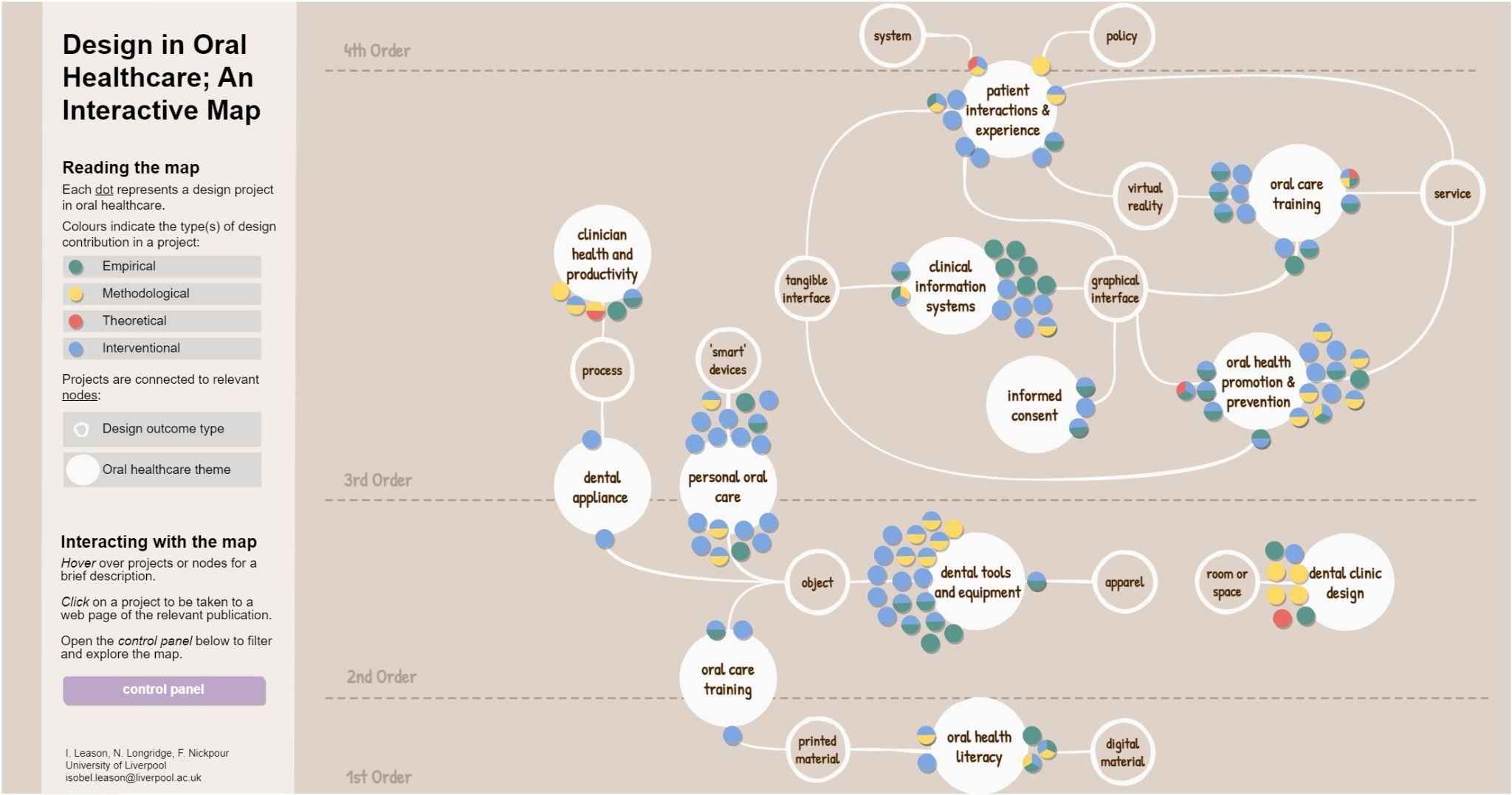

Supplement: Supplementary file 1 — Appendix S1. Supporting Information [file CDOE-52-1-s001.docx]
